# Data Assimilation by Stochastic Ensemble Kalman Filtering to Enhance Turbulent Cardiovascular Flow Data From Under-Resolved Observations

**DOI:** 10.3389/fcvm.2021.742110

**Published:** 2021-11-02

**Authors:** Dario De Marinis, Dominik Obrist

**Affiliations:** ^1^ARTORG Center for Biomedical Engineering Research, University of Bern, Bern, Switzerland; ^2^Dipartimento di Meccanica, Matematica e Management and Centro di Eccellenza in Meccanica Computazionale, Politecnico di Bari, Bari, Italy

**Keywords:** data assimilation, Ensemble Kalman Filter, cardiovascular flow, turbulence, ensemble averaging

## Abstract

We propose a data assimilation methodology that can be used to enhance the spatial and temporal resolution of voxel-based data as it may be obtained from biomedical imaging modalities. It can be used to improve the assessment of turbulent blood flow in large vessels by combining observed data with a computational fluid dynamics solver. The methodology is based on a Stochastic Ensemble Kalman Filter (SEnKF) approach and geared toward pulsatile and turbulent flow configurations. We describe the observed flow fields by a mean value and its covariance. These flow fields are combined with forecasts obtained from a direct numerical simulation of the flow field. The method is validated against canonical pulsatile and turbulent flows. Finally, it is applied to a clinically relevant configuration, namely the flow downstream of a bioprosthetic valve in an aorta phantom. It is demonstrated how the 4D flow field obtained from experimental observations can be enhanced by the data assimilation algorithm. Results show that the presented method is promising for future use with *in vivo* data from 4D Flow Magnetic Resonance Imaging (4D Flow MRI). 4D Flow MRI returns spatially and temporally averaged flow fields that are limited by the spatial and the temporal resolution of the tool. These averaged flow fields and the associated uncertainty might be used as observation data in the context of the proposed methodology.

## 1. Introduction

The clinical relevance of 4D Flow Magnetic Resonance Imaging (4D Flow MRI) for quantifying pulsatile and turbulent blood flow in the ascending aorta is limited by spatial and temporal resolution which are in general insufficient for a precise assessment of flow related parameters such as turbulent kinetic energy (TKE), Reynolds shear stress (RSS) and wall shear stress. In recent years, models to overcome these limits have been proposed, e.g., to quantify TKE for assessing aortic stenosis severity (1).

Data Assimilation (DA) can help to enhance the quality of these parameters. In DA, sparse and noisy measurement data (observations) are combined with the forecast solution computed by a numerical (forward) model in order to obtain an improved prediction of the true state of the system. A widely used and well-known technique for DA is the Kalman Filter (KF) proposed by Kalman (2). Computation of an appropriate filter is based on the uncertainties of the observations and of the forward model. These uncertainties can be described by covariances of specific variables that are typically not known *a priori*, such that the appropriate design of a filter requires modeling. The original KF can be applied to a wide range of applications if the physical system can be described by a linear model and data observed from measurements are affected by normally distributed noise. In this context, the forecast computed by the linear model is perturbed by the error of the model, and the forecast mean depends linearly only on the previous time step since the error has zero mean. The covariance of the error is not zero, therefore it also appears in the forecast covariance matrix computation.

In nonlinear systems, the forecast mean and covariance cannot be calculated directly from the previous time step anymore, because of the nonlinear nature of the model operator. This limitation led to enhancements of the basic KF theory. A first example is the Extended Kalman Filter (3) which linearizes the original nonlinear dynamics around the previous state estimates. The ensemble KF (EnKF) (4) replaces the forecast covariance matrix by a sample covariance and estimates the forecast mean and covariance from an ensemble of states of system, which represents the evolution of the state probability density function. This makes the EnKF an important DA tool for ensemble forecasting. If the observations can be interpreted as the result of an ensemble of samples, the noise covariance is replaced by the sample covariance which results in the Stochastic Ensemble Kalman Filter (SEnKF) (5, 6) also known as Ensemble Kalman Filter with perturbed observations.

Many examples of KF techniques applied to fluid dynamics problems have been reported in the literature. Hœpffner et al. (7) used a KF based on the linearized Navier–Stokes equations to reconstruct the relevant statistics of the initial conditions in transitional wall-bounded flow systems. The study has been extended to turbulent wall-bounded flows to estimate the mean turbulent flow profile in the near-wall region by using noisy observations on the wall (8) and to capture the turbulent flow state at the outer boundary of the buffer region of turbulent boundary layer by using an EnKF (9). Gu and Oliver (10) proposed an EnKF to investigate multiphase flows in porous media. Harlim and Majda (11) compare different methods for filtering sparsely observed turbulent geophysical flows in the atmosphere and ocean regimes. Suzuki (12) developed a hybrid unsteady-flow simulation technique combining particle tracking velocimetry (PTV) and direct numerical simulation (DNS) by using a reduced-order KF. Recently, a reduced-order model based on KF has been proposed for turbulent flow configurations showing a successful improvement of the prediction of turbulent features even when the observation is provided only in a limited region (13).

DA using methods different from KF have been also applied to fluid dynamics problems. A variational DA technique based on the minimization of the error between observations and numerical solution in the context of Reynolds Averaged Navier–Stokes (RANS) equations has been applied successfully to reconstruct the mean flow field around a cylinder in laminar regime (14). The variational DA technique has been used to combine mean velocity from 2D PIV observations of flow over an idealized airfoil and a numerical solver for RANS Equation (15).

Both variational and KF-based DA techniques for computational fluid dynamics have been reviewed and compared for unsteady viscous flow applications (16). These techniques are powerful tools, because the coupling of observed data with computational models can remove errors which cannot be identified by using only one of these scientific methodologies alone. However, reliable tools for accurate prediction of complex flow configurations are still lacking.

In the field of cardiovascular flows, DA and Kalman filter have been used in order to improve the accuracy and reliability of physical modeling and to reduce the uncertainty due to the lack of information about boundary conditions, patient-specific geometries and blood viscosity. Gaidzik et al. (17) improved hemodynamic flow prediction by merging Phase-Contrast MRI data with CFD simulations for an idealized aneurysm model where well-controlled laminar flow can be obtained. Canuto et al. (18) implemented an EnKF for the purpose of estimating parameters in cardiovascular models, i.e., a fully zero-dimensional model of the right heart and pulmonary circulation and a coupled 0D–1D model of the lower leg, through the assimilation of clinical measurements of specific patients. DeVault et al. (19) proposed a model for the blood flow in a vital subnetwork of the cerebral vasculature, namely the Circle of Willis. In this model the parameters of the outflow conditions were calibrated using a subset of clinical measurements through EnKF techniques. Arnold et al. (20) used EnKF to estimate the inlet flow waveform in patient-specific arterial network models. Habibi et al. (21) used a reduced-order modeling Kalman filter to provide blood flow data that were more accurate than the computational and synthetic voxel-based experimental datasets with the aim of improving near-wall hemodynamics quantification. In their recent review, Arzani and Dawson (22) present and compare different variational and KF-based DA for modeling cardiovascular flows. Some other studies on merging CFD and 4D flow MRI data using data assimilation are reported here for completeness (23–27).

The aim of this paper is to propose a DA methodology based on the SEnKF approach that can be used to enhance the spatial and temporal resolution of voxel-based flow observations of turbulent pulsatile flow (as in 4D Flow MRI) to improve the assessment of turbulent blood flow in large vessels. To the best of the authors' knowledge, the current work seems to be the first study that tackles turbulence modeling in blood flow with KF-based DA techniques. We propose to consider voxel-based observed data as the result of a volumetric averaging of the true state over the voxel size. The associated sample covariance will take into account the presence of turbulence as well as the noise. The present problem of DA could also be addressed by the 4D-Var method (28, 29) or the Ensemble Kalman Smoother (30) which cope with problems presenting nonlinearity and strong sensitivity to initial conditions as in turbulent flows. Here, we only aim at finding the correct covariance matrix of these fluctuations rather than trying to predict exact, instantaneous fluctuations. The sensitivity to initial conditions ensures that every simulated pulse results in a slightly different realization of the turbulent flow field. The average of this ensemble of flow realizations corresponds to the mean flow in the Reynolds decomposition and the covariance matrix then corresponds to the Reynolds stress tensor. We will apply the SEnKF method mainly for these analogies with the Reynolds decomposition of turbulent flows.

The used forward model comprises a high-order finite-difference flow solver for the Navier–Stokes equations for the Direct Numerical Simulation (DNS) of turbulent incompressible flow (31) which is thoroughly validated and has been used for several complex flow configurations (32–34) and recently for the study of fluid-structure interaction problems (35, 36). The numerical forecast provided by the forward model are decomposed in an expectation value (ensemble-average) and its fluctuations. Such expectation values can be interpreted as a RANS flow field and the associated covariance matrix as the Reynolds stresses (RSS). In that spirit, our SEnKF algorithm applies a correction to the numerical forecast based on its covariance (RSS) and on a set of observed data and their associated covariances.

The remainder of this paper is organized as follows: the section 2 provides a brief description of the theoretical background of turbulent flows, EnKF and SEnKF approaches, and the proposed DA methodology that will estimate the enhanced flow fields. In the section 3, the proposed DA methodology is applied to three cases: Unsteady flow past a circular cylinder confined in a channel; Wall-bounded turbulent flow in a channel; Flow downstream of an aortic valve. The paper concludes with a discussion on the accuracy, efficiency, and versatility of the DA methodology based on these results.

## 2. Methods

### 2.1. Ensemble Averaging

We model incompressible flow of a homogeneous Newtonian fluid with the Navier–Stokes equations


(1) $$\begin{array}{l} \vec{\nabla }\cdot \vec{u}\left(\vec{x},t\right)=0, \end{array}$$

(2) $$\begin{array}{l} \frac{\partial \vec{u}\left(\vec{x},t\right)}{\partial t}+\left(\vec{u}\left(\vec{x},t\right)\cdot \vec{\nabla }\right)\vec{u}\left(\vec{x},t\right)+\frac{1}{\rho }\vec{\nabla }p\left(\vec{x},t\right)-\nu \nabla^{2}\vec{u}\left(\vec{x},t\right)\\ \;-\vec{f}\left(\vec{x},t\right)=0, \end{array}$$

where $\vec{x}=\left\{x,y,z\right\}$ are Cartesian coordinates, and *t* is the time. The variables ρ and ν are the fluid density and the kinematic viscosity, respectively; *p* is the fluid pressure; $\vec{u}$ and $\vec{f}$ are the fluid velocity and the volumetric forcing, respectively.

In the case of turbulent flows, the pressure and velocity fields present chaotic, unsteady changes due to the nonlinear nature of the system and exhibit strong sensitivity to the initial conditions. In practice, this leads to non-reproducible flow fields despite the deterministic nature of the Navier–Stokes (Equation 2) and a statistical approach is often used to study turbulent flow systems. Therefore, we will not aim at predicting exact turbulent fluctuations from noisy observations. Rather, we will formulate a data assimilation scheme to predict statistical properties of the turbulent flow comprising the mean flow and the second moment of the turbulent fluctuations.

The expectation value of a specific turbulent flow quantity may be estimated from an ensemble of multiple realizations of the flow field, i.e., multiple states-of-system. Each realization *r* of the flow field $\vec{u}{\left(\vec{x},t\right)}_{\left(r\right)}$ can be decomposed in an ensemble-averaged field $\langle \vec{u}\left(\vec{x},t\right)\rangle$ and the fluctuations $\vec{u^{\prime }}{\left(\vec{x},t\right)}_{\left(r\right)}$ according to the Reynolds decomposition


(3) $$\begin{array}{l} \vec{u}{\left(\vec{x},t\right)}_{\left(r\right)}=\langle \vec{u}\left(\vec{x},t\right)\rangle +\vec{u^{\prime }}{\left(\vec{x},t\right)}_{\left(r\right)}, \end{array}$$

with the ensemble-average on the *s* states-of-system defined as


(4) $$\begin{array}{l} \langle \vec{u}\left(\vec{x},t\right)\rangle :=\underset{s\to \infty }{\lim}\frac{1}{s}\overset{s}{\underset{r=1}{\sum }}\vec{u}{\left(\vec{x},t\right)}_{\left(r\right)}. \end{array}$$

This leads to the following Reynolds-averaged Navier–Stokes (RANS) equations for incompressible flows:


(5) $$\begin{array}{l} \vec{\nabla }\cdot \langle \vec{u}\left(\vec{x},t\right)\rangle =0 \end{array}$$

(6) $$\begin{array}{l} \frac{\partial \langle \vec{u}\left(\vec{x},t\right)\rangle }{\partial t}+\left(\langle \vec{u}\left(\vec{x},t\right)\rangle \cdot \vec{\nabla }\right)\langle \vec{u}\left(\vec{x},t\right)\rangle +\frac{1}{\rho }\vec{\nabla }\langle p\left(\vec{x},t\right)\rangle \\ \;-\nu \nabla^{2}\langle \vec{u}\left(\vec{x},t\right)\rangle -\langle \vec{f}\left(\vec{x},t\right)\rangle \\ \;=-\vec{\nabla }\cdot \langle \vec{u^{\prime }}\left(\vec{x},t\right)\vec{u^{\prime }}{\left(\vec{x},t\right)}^{\text{T}}\rangle . \end{array}$$

Equations (5) and (6) have the same form as the original (Equations 1, 2) except for the additional term on the right-hand side of Equation (6) comprising the so-called Reynolds Stresses (RSS) $-\rho \langle \vec{u^{\prime }}\left(\vec{x},t\right)\vec{u^{\prime }}{\left(\vec{x},t\right)}^{\text{T}}\rangle$.

In pulsatile flows, the flow configurations are repeated with period *T*, and a generic time *t*_*n*_ can be written as


(7) $$\begin{array}{l} t_{n}=t_{\left(r\right),\phi }=t_{\phi }+\left(r-1\right)T, \end{array}$$

where *t*_ϕ_ is the phase time and *r* is the number of the pulse. The description of the pulsatile dynamics can be reduced to the study of the flow configurations of the basic pulse period. Nonetheless, in presence of turbulence, the periodically subsequent configurations of the pulsatile flow will show differences due to the turbulent fluctuations. Information about the turbulent dynamics of pulsatile flows can be captured decomposing the flow field by phase-averaging, that is obtained by building the ensemble of states-of-system from the periodically subsequent configurations. Thus, the ensemble definition (Equation 4) assumes the following form


(8) $$\begin{array}{l} {\langle \vec{u}\left(\vec{x},t_{n}\right)\rangle }_{\phi }:=\underset{s\to \infty }{\lim}\frac{1}{s}\overset{s}{\underset{r=1}{\sum }}\vec{u}\left(\vec{x},t_{\phi }+\left(r-1\right)T\right) \end{array}$$

where 〈·〉_ϕ_ is the phase-average operation on the periodically subsequent configurations related to the basic time *t*_ϕ_. The pulsatile turbulent dynamics is then described at each basic time *t*_ϕ_ by the phase-averaged flow field ${\langle \vec{u}\left(\vec{x},t_{\phi }\right)\rangle }_{\phi }$ and its covariance ${\langle \vec{u^{\prime }}\left(\vec{x},t_{\phi }\right)\vec{u^{\prime }}{\left(\vec{x},t_{\phi }\right)}^{\text{T}}\rangle }_{\phi }$.

In turbulent configurations presenting a statistically-steady behavior, a time-average corresponds to an ensemble-average. Thus, the ensemble definition (Equation 4) assumes the following form


(9) $$\begin{array}{l} {\langle \vec{u}\left(\vec{x},t\right)\rangle }_{t}:=\underset{s\to \infty }{\lim}\frac{1}{s}\overset{s}{\underset{r=1}{\sum }}\vec{u}\left(\vec{x},t_{0}+\left(r-1\right)\Delta \tau \right) \end{array}$$

where 〈·〉_*t*_ is the time-average operation, *t*_0_ is the initial time value and Δτ a chosen time increment. The statistically-steady turbulent dynamics is then described by the time-averaged flow field ${\langle \vec{u}\left(\vec{x},t\right)\rangle }_{t}$ and its covariance ${\langle \vec{u^{\prime }}\left(\vec{x},t\right)\vec{u^{\prime }}{\left(\vec{x},t\right)}^{\text{T}}\rangle }_{t}$. Note that the definition of the time-average operation (Equation 9) is formally identical to the definition ensemble average (Equation 8) if the number of phases describing the flow is equal to 1 and Δτ corresponds the time period *T*.

In spatially-homogeneous turbulent flows the turbulent features are statistically the same in the flow domain such that the ensemble-average (Equation 4) can be replaced by a volume-average,


(10) $$\begin{array}{l} \bar{\vec{u}\left(\vec{x},t\right)}:=\frac{1}{V}\underset{V}{∭}\vec{u}\left(\vec{\xi },t\right)\text{d}\vec{\xi } \end{array}$$

where $\bar{\;\cdot \;}$ stands for the volume-average operation over the volume *V*. The spatially-homogeneous turbulent dynamics is then described by the volume-averaged flow field $\bar{\vec{u}\left(\vec{x},t\right)}$ and its associated covariance $\bar{\vec{u^{\prime }}\left(\vec{x},t\right)\vec{u^{\prime }}{\left(\vec{x},t\right)}^{\text{T}}}$.

In summary, the ensemble used for statistical characterization of a turbulent flow can be built in different ways depending on the physical behavior of the turbulent flow configuration (statistically-steady, periodic, spatially-homogeneous) leading to different forms of Equation (4). Equations (8)–(10) can be seen as special cases of the following most general form of the ensemble definition Equation (4)


(11) $$\begin{array}{l} {\langle \bar{\vec{u}\left(\vec{x},t_{n}\right)}\rangle }_{\phi }:=\underset{s\to \infty }{\lim}\frac{1}{s}\overset{s}{\underset{r=1}{\sum }}\bar{\vec{u}\left(\vec{x},t_{\left(r\right),\phi }\right)}. \end{array}$$

### 2.2. Stochastic Ensemble Kalman Filtering Approach

The SEnKF approach is used to estimate the state of the system by filtering an ensemble forecast with observations over time. Hereafter, a brief introduction to KF algorithms and then to the SenKF is presented. Details of the algorithms are available in the original papers (2, 4, 5).

The true state-of-system at time *t*_*n*_ is denoted by the state-vector ${\vec{u}}_{n}$. The observation of this state is denoted by the vector ${\vec{d}}_{n}$ which depends on ${\vec{u}}_{n}$ through the observation operator **H** that describes the measurement tool used for the acquisition of the data, such that


(12) $$\begin{array}{l} {\vec{d}}_{n}=\text{H}{\vec{u}}_{n}+{\vec{r}}_{n}, \end{array}$$

where ${\vec{r}}_{n}$ is the observation noise described by a normal distribution ${\vec{r}}_{n}\sim N\left(\vec{0},{\text{R}}_{n}\right)$ with zero mean and covariance **R**_*n*_. The forward model is defined through the operator **M**_*n*_ such that


(13) $$\begin{array}{l} {\vec{u}}_{n}={\text{M}}_{n}{\vec{u}}_{n-1}+{\vec{q}}_{n}, \end{array}$$

where the error ${\vec{q}}_{n}$ follows a normal distribution ${\vec{q}}_{n}\sim N\left(\vec{0},{\text{Q}}_{n}\right)$ with zero mean and covariance **Q**_*n*_. The operator **M**_*n*_ models the physical behavior of the system and can be of linear or nonlinear nature.

To estimate the state at time level *n*, we assume that the state of the system ${\vec{u}}_{n-1}$ given all past observations to that time step ${\vec{d}}_{1:n-1}$ follows a normal distribution with mean ${\vec{\mu }}_{n-1}^{a}$ and with covariance ${\text{P}}_{n-1}^{a}$, such that


(14) $$\begin{array}{l} {\vec{u}}_{n-1}|{\vec{d}}_{1:n-1}\sim N\left({\vec{\mu }}_{n-1}^{a},{\text{P}}_{n-1}^{a}\right). \end{array}$$

Starting from this assumption on the previous time level *n* − 1, we can estimate a forecast of the state vector ${\vec{u}}_{n}$ as


(15) $$\begin{array}{l} {\vec{u}}_{n}|{\vec{d}}_{1:n-1}\sim N\left({\vec{\mu }}_{n}^{f},{\text{P}}_{n}^{f}\right), \end{array}$$

where the superscript *f* stands for the *forecast* step that is the prior update obtained from the forward model. In the following, the superscript *a* denotes the *analysis* step that is the posteriori update of the system obtained by taking into account also the observations at that time step.

The basic idea of the EnKF algorithms (4) is to estimate ${\vec{\mu }}_{n}^{f}$ and ${\text{P}}_{n}^{f}$ from an ensemble of *s* states ${\vec{u}}_{\left(r\right),n}^{f}$ with *r* = 1, 2, …, *s*,


(16) $$\begin{array}{l} {\vec{u}}_{\left(r\right),n}^{f}=\text{M}\left({\vec{u}}_{\left(r\right),n-1}^{a},t_{n}\right) \end{array}$$

(17) $$\begin{array}{l} {\vec{\mu }}_{n}^{f}=\frac{1}{s}\overset{s}{\underset{r=1}{\sum }}{\vec{u}}_{\left(r\right),n}^{f} \end{array}$$

(18) $$\begin{array}{l} {\text{P}}_{n}^{f}=\frac{1}{s}\overset{s}{\underset{r=1}{\sum }}\left({\vec{u}}_{\left(r\right),n}^{f}-{\vec{\mu }}_{n}^{f}\right){\left({\vec{u}}_{\left(r\right),n}^{f}-{\vec{\mu }}_{n}^{f}\right)}^{\text{T}}. \end{array}$$

If the observations ${\vec{d}}_{n}$ can be treated as the result of an ensemble of *s* samples ${\vec{d}}_{\left(r\right),n}$, the observation noise covariance is replaced by a sample covariance, which leads to the following relations for the SEnKF algorithm (5):


(19) $$\begin{array}{l} {\vec{d}}_{\left(r\right),n}=\text{H}{\vec{u}}_{\left(r\right),n}+{\vec{r}}_{\left(r\right),n} \end{array}$$

(20) $$\begin{array}{l} {\vec{d}}_{n}=\frac{1}{s}\overset{s}{\underset{r=1}{\sum }}{\vec{d}}_{\left(r\right),n} \end{array}$$

(21) $$\begin{array}{l} {\text{R}}_{n}=\frac{1}{s}\overset{s}{\underset{r=1}{\sum }}\left({\vec{d}}_{\left(r\right),n}-{\vec{d}}_{n}\right){\left({\vec{d}}_{\left(r\right),n}-{\vec{d}}_{n}\right)}^{\text{T}}. \end{array}$$

After the prior update obtained from the forecast step (Equations 16–18), the SEnKF algorithms proceeds with the analysis step which updates the state-vector using the observations:


(22) $$\begin{array}{l} {\vec{u}}_{\left(r\right),n}^{a}={\vec{u}}_{\left(r\right),n}^{f}+{\text{K}}_{n}\left({\vec{d}}_{\left(r\right),n}-\text{H}{\vec{u}}_{\left(r\right),n}^{f}\right) \end{array}$$

(23) $$\begin{array}{l} {\vec{\mu }}_{n}^{a}=\frac{1}{s}\overset{s}{\underset{r=1}{\sum }}{\vec{u}}_{\left(r\right),n}^{a}, \end{array}$$

(24) $$\begin{array}{l} {\text{P}}_{n}^{a}=\frac{1}{s}\overset{s}{\underset{r=1}{\sum }}\left({\vec{u}}_{\left(r\right),n}^{a}-{\vec{\mu }}_{n}^{a}\right){\left({\vec{u}}_{\left(r\right),n}^{a}-{\vec{\mu }}_{n}^{a}\right)}^{\text{T}}. \end{array}$$

Equation (22) defines the correction of the forecast ${\vec{u}}_{\left(r\right),n}^{f}$ to the state estimate ${\vec{u}}_{\left(r\right),n}^{a}$ through the so-called Kalman gain **K**_*n*_ which depends on the observation uncertainty **R**_*n*_ and the uncertainty of the forecast estimate ${\text{P}}_{n}^{f}$ according to


(25) $$\begin{array}{l} {\text{K}}_{n}={\text{P}}_{n}^{f}{\text{H}}^{\text{T}}{\left(\text{H}{\text{P}}_{n}^{f}{\text{H}}^{\text{T}}+{\text{R}}_{n}\right)}^{-1}. \end{array}$$

For the limiting case, where the observations are assumed to be perfect, i.e., **H**_*n*_ = **I** and **R**_*n*_ → **0** (where **I** is the identity matrix), the Kalmain gain reduces to **K**_*n*_ = **I** such taht the analysis step becomes ${\vec{u}}_{\left(r\right),n}^{a}={\vec{d}}_{\left(r\right),n}$. This limiting case illustrates that the state estimate ${\vec{u}}_{\left(r\right),n}^{a}$ tends to be dominated by the observations ${\vec{d}}_{\left(r\right),n}$ if **R**_*n*_ is small. In contrast, **K**_*n*_ → **0** if the prior covariance ${\text{P}}_{n}^{f}$ tends to zero, i.e., if the level of forecast certainty is high. In that case, the filter ignores the observations and ${\vec{u}}_{\left(r\right),n}^{a}\approx {\vec{u}}_{\left(r\right),n}^{f}$.

### 2.3. Data Assimilation Methodology for Pulsatile Turbulent Flows

In the following, we extend the SEnKF approach to formulate a DA methodology for pulsatile, turbulent flows.

#### 2.3.1. Data Acquisition

In the present context, we assume that observations of pulsatile, turbulent flows are available as voxel-based data with voxel size *h*. Often small turbulent length scales cannot be captured by such observations. The measured data can be seen as the result of a volumetric average over the voxel volume according to Equation (10). Moreover, if the observations are run for *p* repetitive pulses, the voxel data can also be phase averaged to obtain estimates of mean and a covariance according to Equation (11). Each observed voxel(*v*)-based data ${\vec{d}}_{v;\left(s\right),\phi }$ measured in the pulse *s* has an error ${\vec{r}}_{v;\left(s\right),\phi }$ whose covariance will be considered as the result of a voxel-phase-average. This leads to the following definitions of the data ${\vec{d}}_{v;\left(s\right),\phi }$, the associated mean ${\vec{d}}_{v;\phi }$ and sample covariance **r**_*v*; ϕ_:


(26) $$\begin{array}{l} {\vec{d}}_{v;\left(s\right),\phi }=\bar{\vec{u}\left(\vec{x},t_{\left(s\right),\phi }\right)} \end{array}$$

(27) $$\begin{array}{l} {\vec{d}}_{v;\phi }={\langle \bar{\vec{u}\left(\vec{x},t_{\phi }\right)}\rangle }_{\phi }=\frac{1}{p}\overset{p}{\underset{s=1}{\sum }}{\vec{d}}_{v;\left(s\right),\phi }, \end{array}$$

(28) $$\begin{array}{l} {\text{r}}_{v;\phi }={\langle \bar{\vec{u}\left(\vec{x},t_{\phi }\right)}\prime \;{\bar{\vec{u}\left(\vec{x},t_{\phi }\right)}\prime }^{\text{T}}\rangle }_{\phi }\\ \;=\frac{1}{p}\overset{p}{\underset{s=1}{\sum }}\left({\vec{d}}_{v;\left(s\right),\phi }-{\vec{d}}_{v;\phi }\right){\left({\vec{d}}_{v;\left(s\right),\phi }-{\vec{d}}_{v;\phi }\right)}^{\text{T}}. \end{array}$$

In the classical relation (Equation 21), the covariance matrix is written with respect to the entire state-vector ${\vec{d}}_{n}$. Here for simplicity we assume that covariance between velocities of different voxels is zero; thus, we define the covariance matrix **r**_*v*_ independently for each voxel *v*.

#### 2.3.2. Forecast Solution

The forecast solution is computed using a Navier–Stokes solver for direct numerical simulation (DNS). In the EnKF formalism, this solver represents the nonlinear forward model (**M** = **DNS**) between two subsequent update steps *n* − 1 and *n* such that


(29) $$\begin{array}{l} {\vec{u}}_{n}=\text{DNS}\left({\vec{u}}_{n-1},t_{n}\right)+{\vec{q}}_{n}, \end{array}$$

(30) $$\begin{array}{l} {\vec{u}}_{\left(r\right),n}^{f}=\text{DNS}\left({\vec{u}}_{\left(r\right),n-1}^{a},t_{n}\right). \end{array}$$

The Kalman time step Δ*t*_*KF*_ = *t*_*n*+1_ − *t*_*n*_ between two subsequent updates should not be confused with the DNS time step of the Navier–Stokes solver. Even though the DNS Equation 30 returns a deterministic solution ${\vec{u}}_{\left(r\right),n}^{f}$ affected only by a numerical error, we know that turbulent flows have a stochastic behavior that can be investigated *a posteriori* by using an ensemble-average approach in order to evaluate the effect of the turbulent fluctuations. The deterministic solution ${\vec{u}}_{\left(r\right),n}^{f}$ differs from the ground-truth solution ${\vec{u}}_{n}$ of Equation 29 because of the error ${\vec{q}}_{n}$ which is composed of three contributions of different nature: a numerical error ${\vec{q}}_{n_{NUM}}$ due to the limited numerical accuracy of the discretized Navier–Stokes equations; an uncertainty ${\vec{q}}_{n_{TUR}}$ due to the stochastic nature of turbulent fluctuations; and a modeling error ${\vec{q}}_{n_{MOD}}$ due to the uncertainty error (e.g., unknown boundary conditions, viscosity) typical of blood flow physical modeling:


(31) $$\begin{array}{l} {\vec{q}}_{n}={\vec{q}}_{n_{NUM}}+{\vec{q}}_{n_{TUR}}+{\vec{q}}_{n_{MOD}}. \end{array}$$

Here, the numerical error ${\vec{q}}_{n_{NUM}}$ is assumed to be negligible, because in a DNS its magnitude must be lower than the magnitude of turbulent fluctuations. In the case of blood flow simulations, the error ${\vec{q}}_{n_{MOD}}$ will add additional uncertainty that will affect the forecast solution.

In the present work, the DNS data ${\vec{u}}_{\left(r\right),n}^{f}$ is considered as a stochastic realization of the ground truth, i.e., it comprises a mean flow component and a random turbulent fluctuation. In the language of the Kalman filter theory, the mean flow is the ground truth and the Reynolds stress tensor is the covariance matrix **P** of the forecast noise. This interpretation neglects the effect of numerical noise and modeling uncertainty. The appropriate choice of the forcast ensemble for the SEnKF algorithm is important for obtaining a good filter for the specific flow configuration. For pulsatile turbulent configurations, we propose to build the ensemble of states from multiple pulses, i.e., each pulse is considered a different realization. First, the pulse period *T* is divided in a number of intervals *N*_Φ_ defined by the basic time *t*_ϕ_ = *t*_1_, *t*_2_, …, *t*_*N*_ϕ__ with *t*_*N*_ϕ__ = *T*. We generate an increasingly large ensemble by running the forward model for multiple pulses. This is a significant deviation from the standard SEnKF concept, where the ensemble is typically built from multiple computations of the forward model with slightly different initial conditions.

In accordance with Equations (16)–(18), the forecast state-vector, its mean and associated sample covariance at each computational grid node *g* are then computed as:


(32) $$\begin{array}{l} {\vec{u}}_{g;\left(s\right),\phi }^{f}\equiv {\vec{u}}^{f}\left({\vec{x}}_{g},t_{\left(s\right),\phi }\right), \end{array}$$

(33) $$\begin{array}{l} {\vec{\mu }}_{g;\left(s\right),\phi }^{f}\equiv {\langle {\vec{u}}^{f}\left({\vec{x}}_{g},t\right)\rangle }_{\left(s\right),\phi }=\frac{1}{s}\overset{s}{\underset{r=1}{\sum }}{\vec{u}}_{g;\left(r\right),\phi }^{f}, \end{array}$$

(34) $$\begin{array}{l} {\langle {\vec{u^{\prime }}}^{f}\left({\vec{x}}_{g},t\right){\vec{u^{\prime }}}^{f}\left({\vec{x}}_{g},t\right)\rangle }_{\left(s\right),\phi }=\frac{1}{s}\overset{s}{\underset{r=1}{\sum }}\left({\vec{u}}_{g;\left(r\right),\phi }^{f}-{\vec{\mu }}_{g;\left(s\right),\phi }^{f}\right)\\ {\left({\vec{u}}_{g;\left(r\right),\phi }^{f}-{\vec{\mu }}_{g;\left(s\right),\phi }^{f}\right)}^{\text{T}}. \end{array}$$

Here for simplicity we assume that covariance between velocities of different grid nodes is zero. Thus, we define the covariance matrix ${\text{p}}_{g}^{f}$ independently for each grid node.

The ensemble will be very small in the beginning and only becomes a statistically useful sample of realizations after several pulses. In the first pulse of the numerical simulation (*s* = 1), the sample covariance ${\text{p}}_{g;\left(1\right),\phi }^{f}$ would be equal to zero according to Equation (34). Therefore, the covariance ${\text{p}}_{g;\left(s\right),\phi }^{f}$ is computed in the following way:


(35) $$\begin{array}{l} {\text{p}}_{g;\left(s\right),\phi }^{f}=\frac{1}{s}\left(D+\overset{s}{\underset{r=1}{\sum }}\left({\vec{u}}_{g;\left(r\right),\phi }^{f}-{\vec{\mu }}_{g;\left(s\right),\phi }^{f}\right){\left({\vec{u}}_{g;\left(r\right),\phi }^{f}-{\vec{\mu }}_{g;\left(s\right),\phi }^{f}\right)}^{\text{T}}\right), \end{array}$$

where *D* is a diagonal matrix whose elements are on order of magnitude larger than the expected covariance one of the specific flow configuration. *D* ensures the positiveness of ${\text{p}}_{g;\left(s\right),\phi }^{f}$ at the beginning of the simulation, and then its influence disappears for increasingly larger ensembles.

#### 2.3.3. Data Assimilation Algorithm

**Figure 1:**
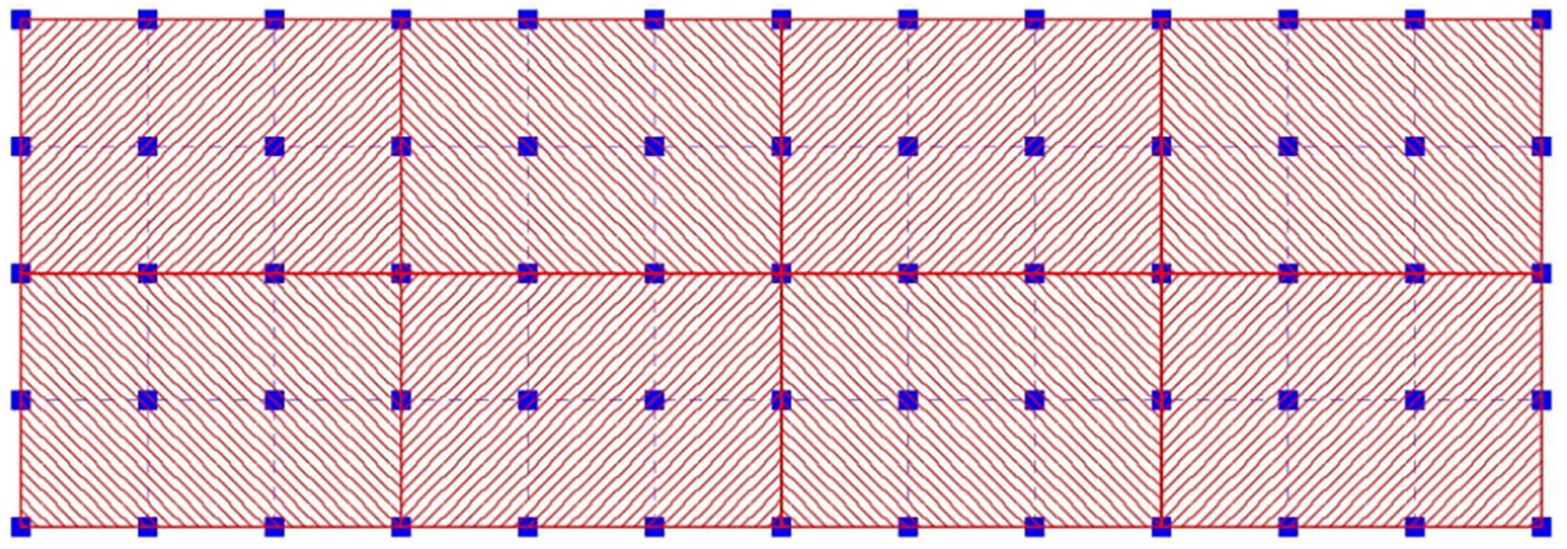
Overlapping of a 2D voxel grid (red) with respect to the 2D computational grid (blue) for *g*_*v*_ = 4 × 3 = 12.

The proposed methodology aims at using observed data on a voxel-grid in combination with the forecast solution computed by DNS on a finer computational grid. To this end, the voxels *m*_*v*_ of the voxel grid are overlapped with the computational grid nodes *m*_*g*_, where the state-vector at each time *t*_*n*_ is defined on each grid node *g* as ${\vec{u}}_{g;n}={\left\{u_{x},u_{y},u_{z}\right\}}_{g;n}$. For Cartesian grids, *g*_*v*_ computational grid-nodes will lie inside each voxel *v*; and it is possible that a grid node *g* belongs to more than one voxel, i.e., the grid node is located exactly on the borders of adjacent *v*_*g*_ voxels (Figure 1).

In the present context, ${\vec{d}}_{v;\left(s\right),\phi }$ is the value that the solution would have on all computational grid nodes *g*_*v*_ lying inside the voxel *v*, if the data ${\vec{d}}_{v;\left(s\right),\phi }$ was constant over the whole voxel volume. It is clear that this is generally not the case. The discrete form of the observation operator **H** that maps the voxel-based data to the grid is a 3*m*_*v*_ × 3*m*_*g*_ matrix assuming the following form:


(36) $$\begin{array}{l} {\left[H_{ij}\right]}_{v,g}=\phi_{v,g}\delta_{ij} \end{array}$$

where δ_*ij*_ is the Kronecker delta, *i*, *j* = 1, 2, 3 and


(37) $$\begin{array}{l} \phi_{v,g}=\{\begin{array}{cc} \frac{1}{g_{v}}, & \text{if }g\text{ lies inside }v,\\ 0, & \text{otherwise.} \end{array} \end{array}$$

Solving the analysis step to update the entire state-vector ${\vec{u}}_{1:m_{g};\left(s\right),\phi }$ requires the computation on huge matrices **P**, **R** and **H** of size 3*m*_*g*_ × 3*m*_*g*_, 3*m*_*v*_ × 3*m*_*v*_, and 3*m*_*v*_ × 3*m*_*g*_, respectively. However, we can exploit the block-structure of **H** comprising of blocks **h** of size 3 × 3*g*_*v*_ which correspond to single voxels. From an algorithmic point of view, this allows to only use *m*_*v*_ dense matrices **h** instead of the huge sparse matrix **H** which dramatically reduces the computational cost. There, the Kalman gain term is written as


(38) $$\begin{array}{l} {\vec{k}}_{g;\left(s\right),\phi }={\sum_{v=1}^{m_{v}}\Psi_{g,v}[{\text{p}}_{1:g_{v};\left(s\right),\phi }^{f}{\text{h}}^{\text{T}}\left(\text{h}{\text{p}}_{1:g_{v};\left(s\right),\phi }^{f}{\text{h}}^{\text{T}}+{\text{r}}_{v,\phi }\right)}^{-1}\\ {\;\left({\vec{d}}_{v;\left(s\right),\phi }-\text{h}{\vec{u}}_{1:g_{v};\left(s\right),\phi }^{f}\right)]}_{g}, \end{array}$$

where Ψ_*g*,1:_*m*__*v*__ are weights for interpolating data between the discrete values of the Kalman gain obtained from the different *m*_*v*_ voxels to the grid node *g*. Since each grid-node solution depends only on the data of the *v*_*g*_ voxels, we define


(39) $$\begin{array}{l} {\text{Ψ}}_{g,v}=\{\begin{array}{cc} \frac{1}{v_{g}}, & \text{if }g\text{ lies inside }v,\\ 0, & \text{otherwise.} \end{array} \end{array}$$

Finally, the updated solution ${\vec{u}}_{\left(s\right),\phi }^{a}$ is computed in the analysis step of the SEnKF as


(40) $$\begin{array}{l} {\vec{u}}_{g;\left(s\right),\phi }^{a}={\vec{u}}_{g;\left(s\right),\phi }^{f}+{\vec{k}}_{g;\left(s\right),\phi }. \end{array}$$

In conclusion, the proposed methodology deals with data obtained by observations on coarse grids, and enhances them by means of the SEnKF algorithm, obtaining a new set of data on finer grids. The entire DA methodology is summarized in the Algorithm 1.

**Algorithm 1:**
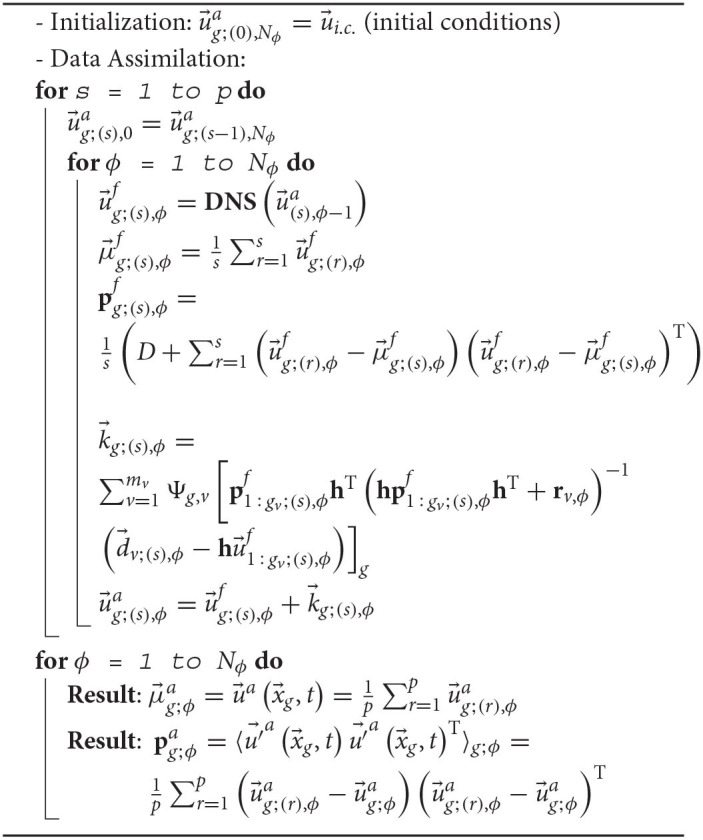
Data Assimilation algorithm for computing enhanced volex-based data

### 2.4. Direct Numerical Simulation

All numerical simulations have been performed using a high-order Navier–Stokes solver for the DNS of incompressible flows. The governing Equations (1) and (2) are discretized on a staggered Cartesian grid using sixth-order finite-difference schemes. A multigrid method is used for solving the Poisson problem and time integration is performed using a three-step Runge–Kutta scheme. Details of the implementation are given in Henniger et al. (31).

The simulations use a combination of periodic boundary conditions and the fringe forcing technique (37) which damps incoming flow disturbances and forces the flow field toward a desired velocity profile by applying a suitable volume force $\vec{f}$ of the right-hand side of the Navier–Stokes (Equation 1). The general form of the fringe forcing is given by


(41) $$\begin{array}{l} \vec{f}\left(\vec{x},t\right)=\lambda \left(\vec{x}\right)\left(\vec{U}\left(\vec{x},t\right)-\vec{u}\left(\vec{x},t\right)\right) \end{array}$$

where $\lambda \left(\vec{x}\right)$ is the fringe function and $\vec{U}\left(\vec{x},t\right)$ is the desired velocity to be imposed. The fringe function is non-zero only within the so-called fringe region which is typically located at the edge of the computational domain. In the physically relevant regions of the DNS, the fringe function is zero.

This technique has been exploited to impose inflow velocity profiles, to model outflow conditions and to model complex geometries by setting $\vec{U}=0$ within immersed objects. The fringe forcing has similarities to the Kalman analysis step (Equation 22), where the Kalman gain *K* acts like a fringe function and the observation $\vec{d}$ corresponds to the desired velocity $\vec{U}$. However, the fringe forcing is added to the right-hand side of a differential equation and drives the solution toward $\vec{u}$ with a time scale 1/λ, whereas the Kalman update is an algebraic equation which nudges the solution toward the observation at a time scale Δ*t*_*KF*_/||*K*||, where Δ*t*_*KF*_ is the time between two subsequent analysis steps.

## 3. Results

In the present study, all observations are available on Cartesian voxel grids and obtained either by experiments or other numerical simulations (synthetic data). In case of synthetic data, the voxel data are extracted from the computational grid, and each voxel comprises multiple *g*_*v*_ grid nodes of the computational grid. The observations have been run for *p* multiple pulses, and the synthetic voxel data are collected in order to obtain a mean and a covariance estimate in accordance with Equation (11). Each voxel data ${\vec{d}}_{v;\left(s\right),\phi }$ computed in the specific pulse *s* has an error ${\vec{r}}_{v;\left(s\right),\phi }$ whose covariance will be considered as the result of a voxel-and-phase-average; Equation (26) assumes the following form:


(42) $$\begin{array}{l} {\vec{d}}_{v;\left(s\right),\phi }\equiv \bar{\vec{u}\left(\vec{x},t_{\left(s\right),\phi }\right)}=\frac{1}{g_{v}}\overset{g_{v}}{\underset{g=1}{\sum }}{\vec{u}}_{g;\left(s\right),\phi }. \end{array}$$

The methodology has been validated for test problems of increasing complexity, starting with a periodic and non-turbulent configuration, then extending the application of the methodology to a turbulent configuration, and finally applying the methodology to a pulsatile and turbulent configuration. Results have been evaluated comparing global flow-related parameters and local profiles with respect to the ground truth and observed data.

The first flow related parameter that has been chosen is the friction Reynolds number *Re*_τ_ which is defined using the friction velocity *U*_τ_,


(43) $$\begin{array}{l} Re_{\tau }=\frac{U_{\tau }L_{ref}}{\nu }=U_{\tau }\frac{Re}{U_{ref}}=Re\;\sqrt{\frac{1}{Re}\frac{L_{ref}}{U_{ref}}\left|\langle \langle \frac{\partial u_{x}\left(\vec{x},t\right)}{\partial y}\rangle \rangle \right|}\;, \end{array}$$

where *Re* is the Reynolds number based on reference values (*U*_*ref*_, *L*_*ref*_) of the flow configuration, the average 〈〈·〉〉 is calculated at the specific time *t* over the wall. The velocity gradient is discretized with a first-order finite difference scheme on the computational grid. Mean velocity and RSS profiles are commonly described in wall units, i.e., by dimensionless variables *y*^+^ and *u*^+^ obtained by normalization with respect to friction velocity *U*_τ_ and flow parameters,


(44) $$\begin{array}{l} u^{+}=\frac{u}{U_{\tau }},\;y^{+}=\frac{yU_{\tau }}{\nu }, \end{array}$$

where *y* is the distance to the wall and *u* is the velocity component parallel to the wall.

The turbulent kinetic energy (TKE) is a second parameter used to quantify the turbulence in the bulk flow. We define TKE within a given volume interest *V*, such that


(45) $$\begin{array}{l} TKE\left(t\right)=\frac{1}{2}\bar{\langle u_{x}^{\prime }{\left(\vec{x},t\right)}^{2}+u_{y}^{\prime }{\left(\vec{x},t\right)}^{2}+u_{z}^{\prime }{\left(\vec{x},t\right)}^{2}\rangle } \end{array}$$

Finally, voxel-and-phase(time)-averaged velocity fields and/or RSS fields are presented for the different configurations.

### 3.1. Periodically Oscillating Flow

**Figure 2:**
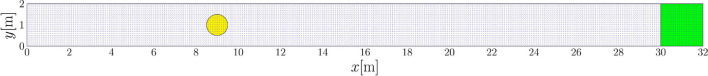
Periodically oscillating flow: configuration of the cylinder confined in the channel. In the green fringe region the inflow is imposed. The cylinder is modeled in the yellow fringe region with $\vec{u}=0$.

The proposed DA methodology is first validated for an unsteady flow past a cylinder confined in a channel (Figure 2). The size of the channel is 32*h* × 2*h* × *h* where *h* = 1 [m] is the channel half-width. The positive *x*-direction is the stream-wise direction, the *y*-axis is perpendicular to the walls at *y* = 0 and *y* = 2*h* and the *z*-axis points in span-wise direction. The diameter of the cylinder is equal to *h* and its center *C* has coordinates (*x, y*) = (9*h, h*) and an axis parallel to the *z*-direction. Periodic boundary conditions have been imposed in both the stream-wise and span-wise direction, whereas a no-slip condition is enforced at the top and bottom wall. Fringe forcing at the end of the domain (green region in Figure 2) is used to enforce plug flow with velocity *U*_*x*_ = 1, *U*_*y*_ = 0, *U*_*z*_ = 0 [m/s] at the inflow (using λ = 50 in Equation 41). The presence of the cylinder is modeled by using an another fringe region with $\vec{U}=0$ and λ = 100 for the grid nodes within the cylinder (yellow region in Figure 2). The combination of the plug flow with the no-slip b.c. will produce a thin boundary layer in the green fringe region. The thickness of this boundary layer will increase and the flow will develop a parabolic profile upstream of the cylinder. The Reynolds number is set equal to 150 based on *h* and the inflow velocity *U*_*x*_.

#### 3.1.1. Ground Truth

**Figure 3:**
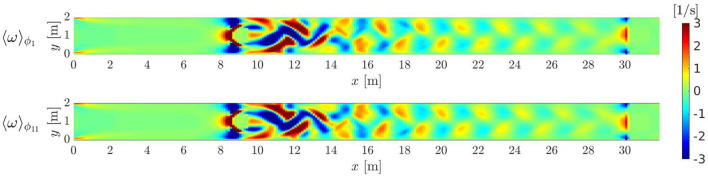
Periodically oscillating flow: vorticity ω fields for two different phase averaging. ϕ_1_ corresponds to basic time *t*_1_ = 0.099755 [s], while ϕ_11_ corresponds to basic time *t*_11_ = 1.097305 [s]. Von Kármán vortex street develops downstream the cylinder.

First, a DNS has been run to obtain the ground truth, using a computational grid of 257 × 33 × 3 grid nodes. At the given Reynolds number, the flow past a confined cylinder presents vortex shedding where the vortices detach periodically with a period of *T*=1.9951 [s] from either side of the body forming a Von Kármán vortex street in the wake of the cylinder (Figure 3).

#### 3.1.2. Data Acquisition

**Figure 4:**
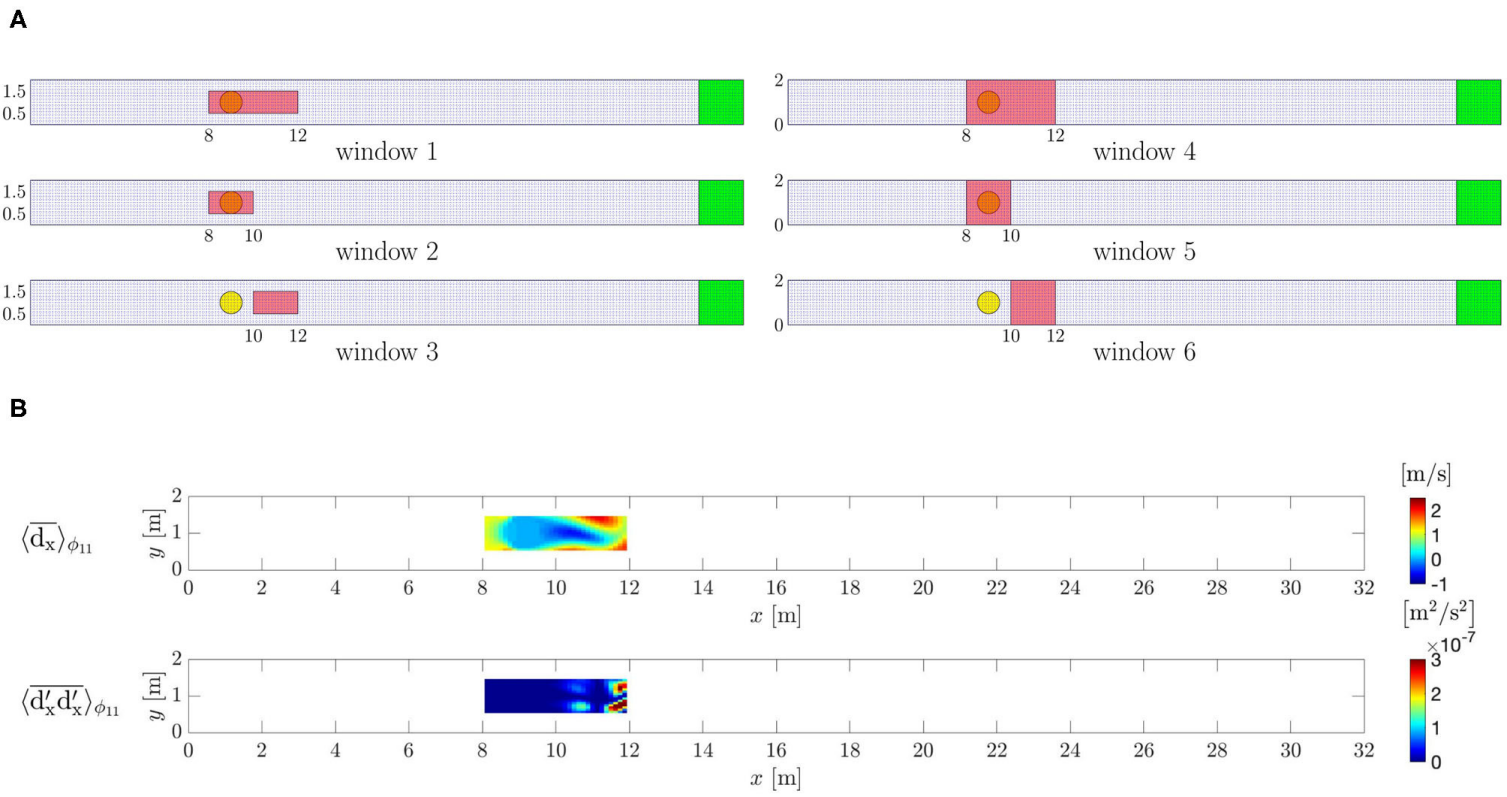
Periodically oscillating flow. **(A)** Windows where the data are extracted from the ground-truth flow field. **(B)** Mean *x*-velocity and associated *xx*- covariance component observed from the ground-truth DNS within window 1.

Second, the observation data ${\vec{d}}_{v;\left(s\right),\phi }$ have been extracted from the ground-truth DNS. The period *T* has been divided in *N*_Φ_ = 20 equal time intervals and the data are collected for *p* = 500 periods starting after 100*T* in order to overcome the initial transient leading to the periodic solution. Six different regions of interest (windows) have been selected to sample the data (Figure 4A). Window 1 includes the cylinder and the downstream region from where the vortex shedding originates. Window 2 and window 3 are obtained as the left and right half of window 1, respectively, to study the capability of the proposed method to reconstruct the flow downstream of the cylinder with and without the data of the cylinder itself. The windows 1, 2, and 3 have the lengths 4, 2, and 2, *h*, respectively. Their height is *h* and they are centered in the *y*-direction and extend over the whole *z*-direction. Extending the windows 1, 2, and 3 to the overall *y*-domain leads to the windows 4, 5, and 6, respectively. The choice of window 4, 5, and 6 allows to assess whether the availability of data close to the channel walls will improve the flow reconstruction by using the DA algorithm or not.

Each ${\vec{d}}_{v;\left(s\right),\phi }$ is computed for a voxel formed by *g*_*v*_ = 2 × 2 × 2 grid nodes by averaging the ground-truth solution of the corresponding *g*_*v*_ nodes. These voxel-averaged values are then further phase-averaged in order to obtain voxel-and-phase averaged data ${\vec{d}}_{v;\phi }$ and the associated covariances **r**_*v*; ϕ_ for all the phases, within the specific window (Figure 4B). Because the flow is not turbulent, the covariance will be almost zero and its value will just include the covariance of the numerical error of the DNS solver and the (very low) inaccuracy of the chosen period value *T*.

#### 3.1.3. Comparison Between Ground Truth and DA Predictions

In the DA predictions only the no-slip b.c. at the walls and the fringe forcing for the inflow are imposed *a priori*. The cylinder fringe region is not used anymore and only the use of the observed data in the SEnKF algorithm, will reconstruct the flow field in the wake of the cylinder. The diagonal matrix **D** in Equation (35) is set to {*D*_*ii*_} = 1, 000. Six DA predictions, i.e., DA 1, DA 2, …DA 6, have been run by using the data set extracted from the six windows of interest, i.e., window 1, window 2, …window 6, respectively.

**Figure 5:**
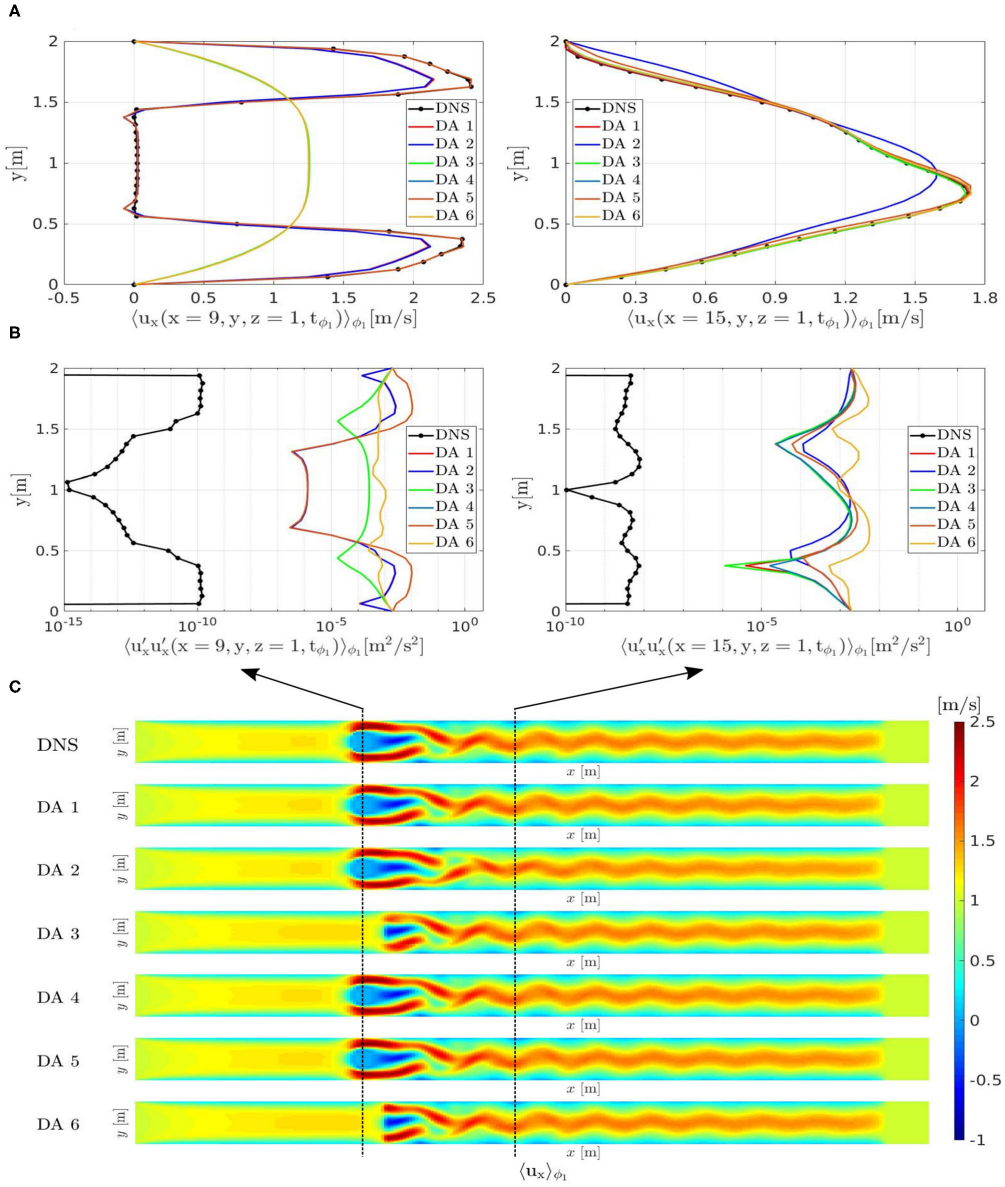
Periodically oscillating flow. Velocity profiles ${\langle u_{x}\left(\vec{x},t_{\phi_{1}}\right)\rangle }_{\phi_{1}}$ **(A)** and *xx*-covariance component profiles ${\langle u_{x}^{\prime }\left(\vec{x},t_{\phi_{1}}\right)u_{x}^{\prime }\left(\vec{x},t_{\phi_{1}}\right)\rangle }_{\phi_{1}}$ **(B)** from DNS and from DA predictions: along a line parallel to the *y*-axis crossing the center of the cylinder at *x* = 9 (left); along a line parallel to the *y*-axis crossing the vortex street at *x* = 15 (right). **(C)** Comparison between the flow field of the ground-truth DNS and of the DA predictions: ${\langle u_{x}\left(\vec{x},t_{\phi_{1}}\right)\rangle }_{\phi_{1}}$.

The voxel-and-phase averaged velocity profiles ${\langle u_{x}\left(\vec{x},t_{\phi }\right)\rangle }_{\phi }$ computed after 500*T* are shown in Figure 5A (left) along a line parallel to the *y*-axis and crossing the center of the cylinder at *x* = 9 [m]. When the data window includes the cylinder (windows 1, 2, 4, 5) the SEnKF forces the flow velocities to zero, because the data inside the cylinder have mean and covariance both equal to zero. In contrast, the DA predictions using data from window 3 and 6 show a nearly parabolic velocity profile, which is the velocity profile that the flow assumes due to the inflow and boundary conditions. This indicates that the proposed methodology has limitations in propagating the information from the observed data in upstream direction. Figure 5A (right) shows the axial velocity profiles at *x* = 15 [m] in the wake of the cylinder: all the DA predictions present good agreement with the ground truth except for the DA prediction performed by using data from window 2. This shows that the window 2 is too small and do not contain enough data to guide the solution of the DA prediction toward the ground truth. Figure 5B shows the *xx*-covariance component profiles at *x* = 15 [m] (right) and at *x* = 9 [m] (left). DA predictions present an error with respect to the ground truth DNS due to the additional uncertainty related to the cylinder boundary condition and to the convergence history of the DA methodology. On the other hand the information is well propagated in downstream direction by the proposed DA methodology as can be seen in Figure 5C for all DA predictions. DA 2 presents a phase-shift with respect to the DNS due to the absence of data in the region surrounding the cylinder which apparently led to a counter-phase vortex detachment.

### 3.2. Turbulent Channel Flow

**Figure 6:**
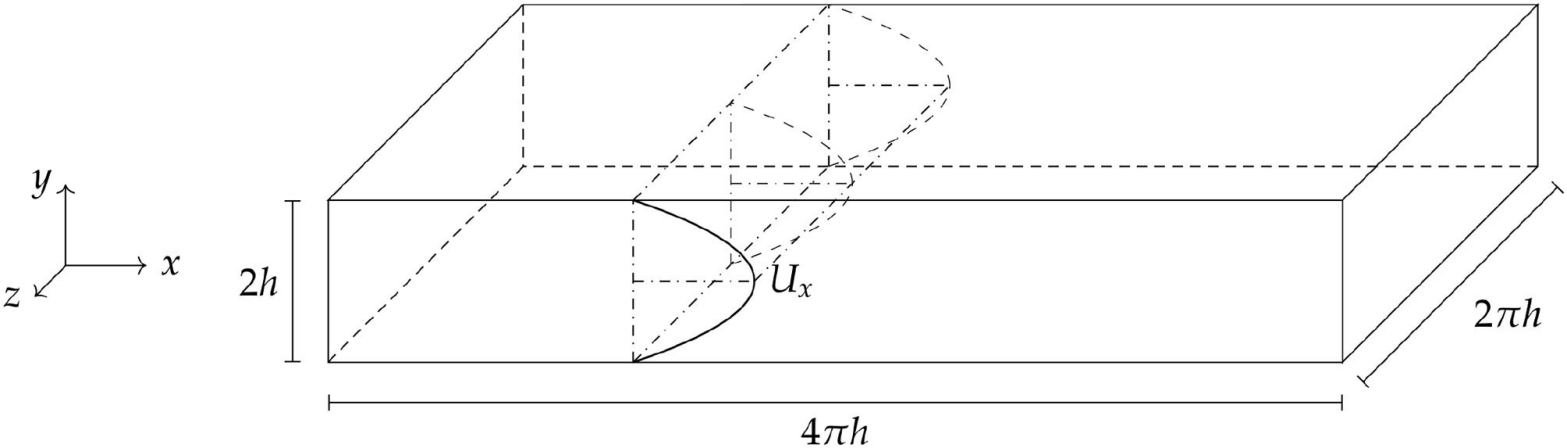
Configuration of the turbulent channel flow. The initial Poiseuille flow has maximum stream-wise velocity *U*_*x*_.

Hereafter, the DA methodology is extended to turbulent flows, and validated for turbulent channel flow. To this end, the dimensions of the channel are set to 4π*h* × 2*h* × 2π*h* according to Kim et al. (38), see Figure 6. The laminar Poiseuille flow $\vec{u}=\left\{\left[y\left(2h-y\right)/h^{2}\right]U_{x},0,0\right\}$ having maximum velocity *U*_*x*_ = 1 [m/s] has been used as initial condition for the velocity field. The Reynolds number (based on the channel half-width *h* = 1 [m] and the initial maximum velocity *U*_*x*_) is set to 5, 000 which is in the range of the intended final application for blood flow in the aorta (39). Periodic boundary conditions have been imposed in both the stream-wise and span-wise direction, while a no-slip condition is ensured at the top and bottom wall. A constant non-dimensional bulk velocity $U_{x}^{bk}=0.667$ [m/s] has been enforced in the stream-wise direction according to Schlatter et al. (40).

#### 3.2.1. Ground Truth

DNS simulations have been run in order to obtain grid-converged solutions that will be considered the ground truth. In order to ensure the transition to turbulence in the channel flow, the initial Poiseuille flow has been perturbed with a two-dimensional (stable) Tollmien–Schlichting (TS) wave with maximum stream-wise velocity amplitude of 3% of the laminar center-line velocity and two superimposed weak oblique (stable) three-dimensional waves with amplitude 0.1% with the same fundamental stream-wise wavelength as the two-dimensional disturbance. The computation of the TS waves was performed using a standard Chebyshev collocation method involving the solution to the Orr–Sommerfeld and Squire equations (41). Sufficient convergence for *Re*_τ_ and *TKE* was achieved for a resolution of 65 × 65 × 65 points.

#### 3.2.2. Data Acquisition

The data ${\vec{d}}_{v;t}$ to be used in the DA predictions have been extracted from the flow field of the ground-truth DNS starting after 200 [s] when a statistically-steady turbulent flow had been established. The ensemble of states-of-system is chosen in accordance with Equation (9).

**Figure 7:**
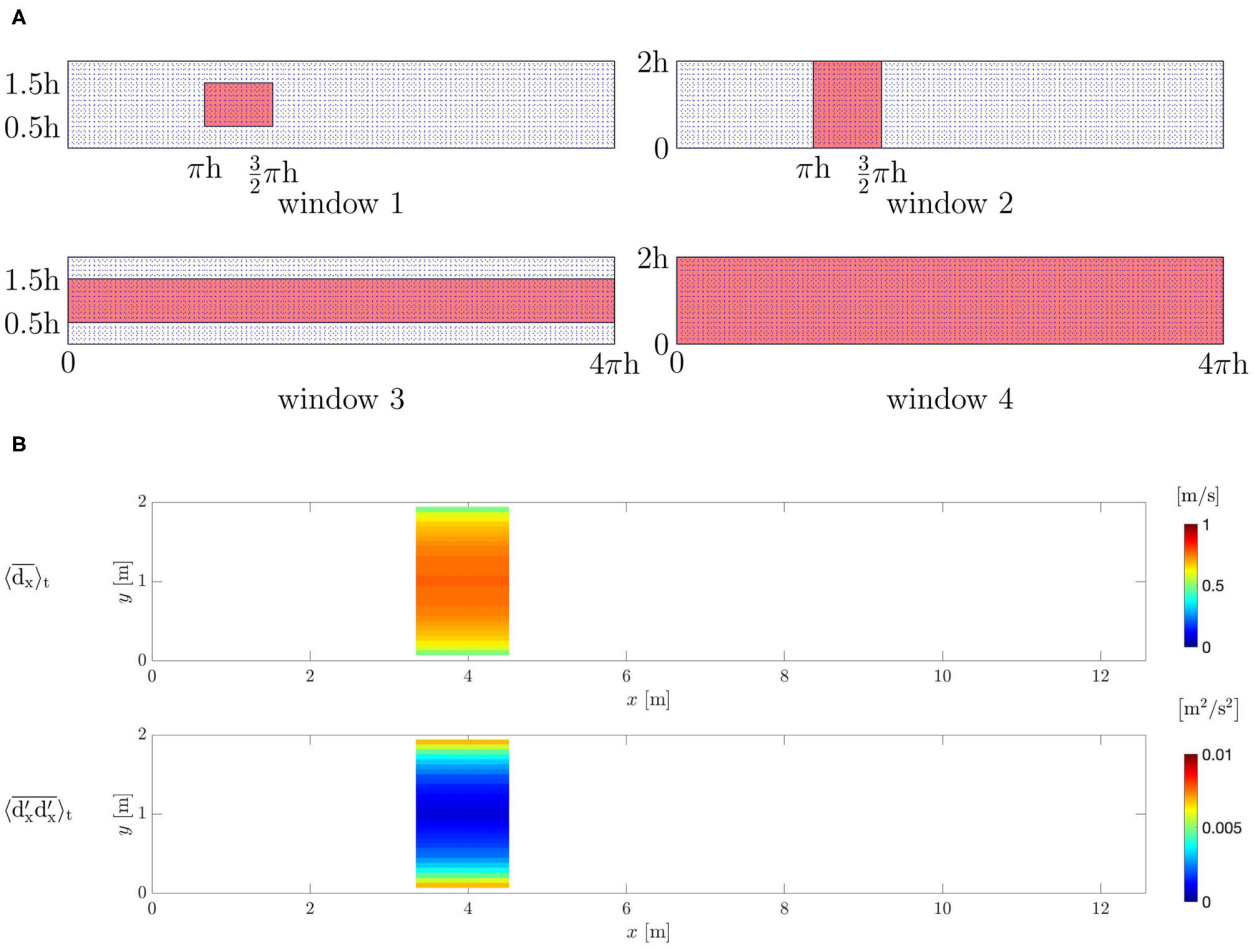
Turbulent channel flow. **(A)** *z*-plane sections of the selected windows in the channel. **(B)** Mean *x*-velocity and associated *xx*- covariance component observed from the ground-truth DNS within window 2.

Four different windows have been selected (Figure 7A). Window 1 has a size of (π/2)*h* in *x*-direction and *h* in *y*-direction. It is centered in *y*-direction. Windows 2 and 3 are obtained by extending the window 1 over the whole *y*- and *x*-direction, respectively, in order to evaluate the influence of the amount of available data on the flow reconstruction. Window 4, which covers the whole domain has been chosen to mimic the configuration of clinical applications where the data are available for the entire blood vessels. All windows are spread over the whole domain in *z*-direction.

Data ${\vec{d}}_{v;t}$ are collected for 4, 000 [s] in order to compute the covariance **r**_*v*; *t*_ in an accurate way onto the voxel-grid. Each ${\vec{d}}_{v;t}$ is obtained by averaging the ground-truth solution over voxels formed by *g*_*v*_ = 3 × 3 × 3 adjacent grid-nodes; these voxel-averaged values are then further time-averaged in order to obtain time-and-space averaged data. Because the flow is turbulent, the covariance will be not zero and its value will include both the covariance of the numerical error of the DNS solver and the turbulent fluctuations covariance (Figure 7B).

#### 3.2.3. Comparison Between Ground Truth and Data Assimilation Predictions

In the DA predictions the contribution of each voxel-based data is spread onto the *g*_*v*_ grid-nodes according to Equation (38). The initial conditions of the velocity are set to Poiseuille flow without any perturbations such that the flow would not show a transition to turbulence without any additional external forcing due to the SEnKF. The diagonal matrix **D** in Equation (35) is set to {*D*_*ii*_} = 10.

Four DA predictions, i.e., DA 1, DA 2, DA 3, DA 4, have been run by using the data set extracted from the four windows of interest, i.e., window 1, window 2, window 3, window 4, respectively.

**Figure 8:**
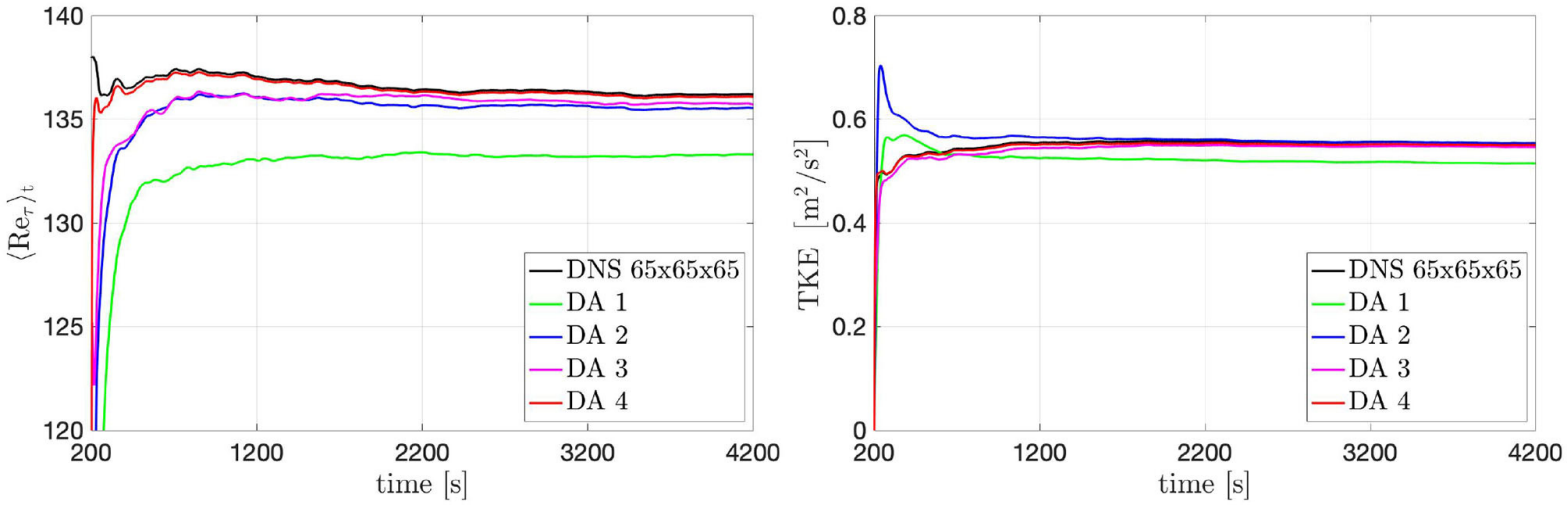
Turbulent channel flow: 〈*Re*_τ_〉_*t*_ (left) and TKE(*t*) (right) evolution over time obtained by using data acquired from different windows.

The results of the DA predictions have been evaluated by comparing the evolution in time of the friction Reynolds number 〈*Re*_τ_〉 and *TKE*(*t*) with the ground truth (Figure 8). At the beginning of the DA predictions, filtering the initial Poiseuille flow by the observed data leads to a transient evolution of the flow to the statistically-steady turbulent configuration. This transient is different for each window with regard to the mean value to which the solution tends and the time required to overcome the transient. After this transient, *Re*_τ_ present the same qualitative behavior in time of the ground truth, even though with different mean value for the different selected data windows. The results are in good agreement with the ground truth, even though they are slightly underestimated. The value of the *TKE* computed in the DA predictions are also good for all configurations. The values of *TKE* stabilize around 0.55 [m^2^/s^2^] for all the DA predictions and the more visible differences with respect to the ground truth are in the amplitude of fluctuations. The best agreement is obtained in DA 4. In contrast, the other data windows lead to a loss of accuracy with respect to the ground truth. DA 1 returns the least accurate trends, and extension of the window in *x*- and *y*-directions, i.e., DA 2 and DA 3, significantly improves accuracy. This means that larger amounts of available data yields DA predictions that tend closer to the ground truth with a shorter numerical transient. For the same reason, DA 4 returns the most accurate predictions.

**Figure 9:**
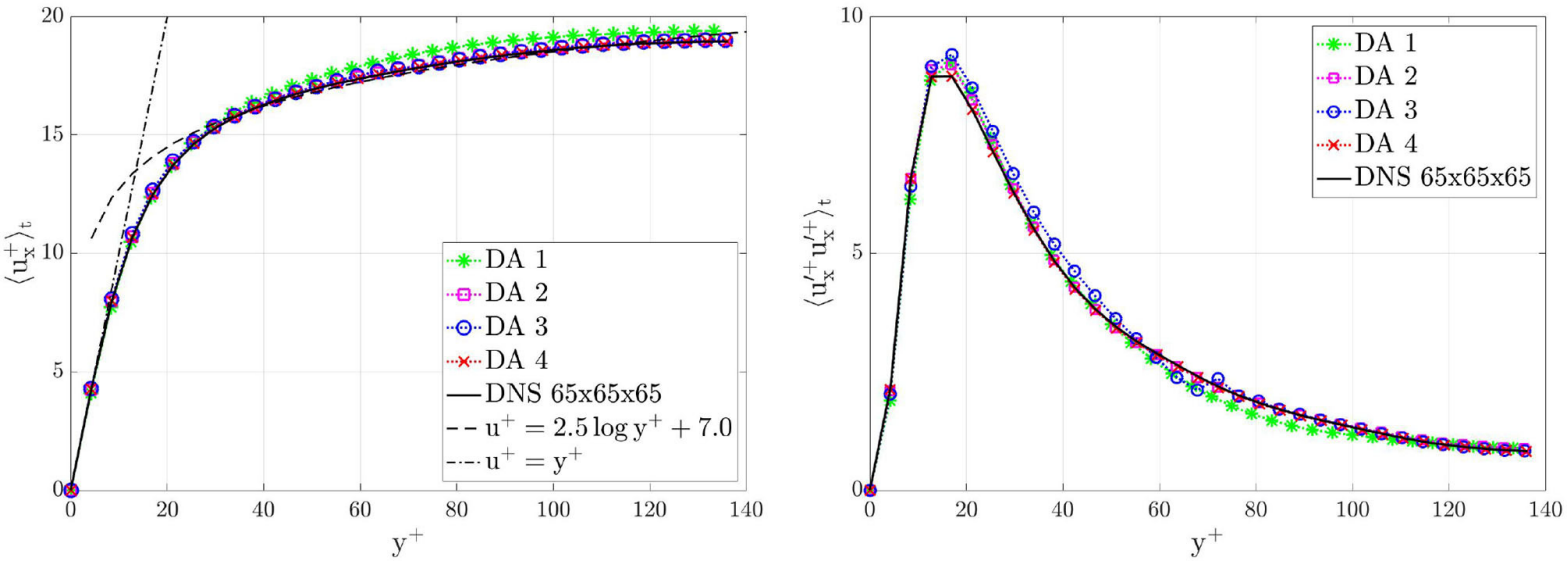
Turbulent channel flow: comparison between DA predictions and ground truth on a line parallel to the *y*-axis and centered in the *z*-direction, i.e., *z* = π*h*, for *x* = 3π*h*. Left: stream-wise mean velocity profiles 〈*u*_*x*_(*x* = 3π*h, y, z* = π*h, t*)〉_*t*_. Right: RSS profiles ${\langle u_{x}^{\prime }\left(x=3\pi h,y,z=\pi h,t\right)u_{x}^{\prime }\left(x=3\pi h,y,z=\pi h,t\right)\rangle }_{t}$. Results are plotted in wall units and are shown for the bottom half part of the channel, i.e., *y* ≤ *h*.

The time-averaged velocity ${\langle u_{x}^{+}\left(\vec{x},t\right)\rangle }_{t}$ profiles computed after 4,000 time units are in almost perfect agreement with the ground-truth profile. Figure 9 shows the comparison between DA prediction and ground truth of the velocity profile ${\langle u_{x}^{+}\left(\vec{x},t\right)\rangle }_{t}$ and the RSS profile ${\langle {u\prime }_{x}^{+}\left(\vec{x},t\right){u\prime }_{x}^{+}\left(\vec{x},t\right)\rangle }_{t}$ on a line parallel to the *y*-axis and centered in the *z*-direction, for *x* = 3π*h*. The DA predictions recover the logarithmic law of the wall *u*^+^ = *Ay*^+^+*B* (42). The precision slightly decreases for DA 1 where not enough data have been used. Moreover, in the boundary region, the RSS profile ${\langle {u\prime }_{x}^{+}\left(\vec{x},t\right){u\prime }_{x}^{+}\left(\vec{x},t\right)\rangle }_{t}$ present different peaks in the different DA predictions. The more data are used in DA predictions, the higher the accuracy is. The loss of accuracy for DA 1 is in agreement with the loss of accuracy of the global parameters evolution shown in the Figure 8.

#### 3.2.4. Downsampling

**Figure 10:**
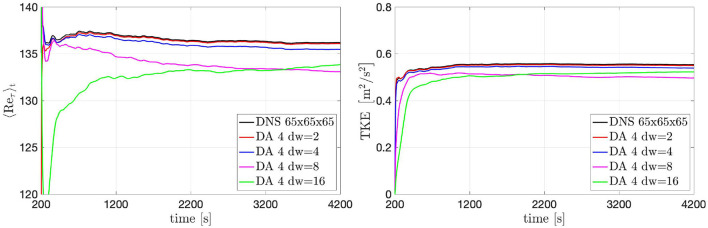
Turbulent channel flow: 〈*Re*_τ_〉_*t*_ (left) and *TKE* (*t*) (right) evolution over time obtained by using data acquired from window 4 with different downsamplings.

In the previous section we evaluated the influence of different window sizes on the accuracy of the results in the DA predictions. Here we want to evaluate the influence of the spatial resolution of the data extracted from the ground-truth solution. To do this, the resolution of the data in window 4 has been down-sampled meaning that the data voxels have a bigger size and the data values are obtained averaging the ground-truth solution over voxels formed by a bigger number of adjacent grid-nodes. The downsampling factor (*dw*) characterizes the resolution of the data acquisition: for instance, a *dw* of 2 means that each ${\vec{d}}_{n}$ is obtained averaging the ground-truth solution over voxels formed by (1 + *dw*) × (1 + *dw*) × (1 + *dw*) adjacent grid-nodes; these voxel-averaged values are then further time-averaged in order to obtain time-and-space averaged data. Here, we analyze the impact of the *dw* on the results starting from the effect on the 〈*Re*_τ_〉_*t*_ and *TKE*(*t*) evolution, see Figure 10. Essentially, a bigger *dw* (lower data resolution) reduce the accuracy of the solution in the DA predictions: this loose of accuracy is due to the. The results obtained with *dw* = 2 and *dw* = 4 are in a very good agreement with the ground-truth results for both 〈*Re*_τ_〉_*t*_ and *TKE*(*t*).

**Figure 11:**
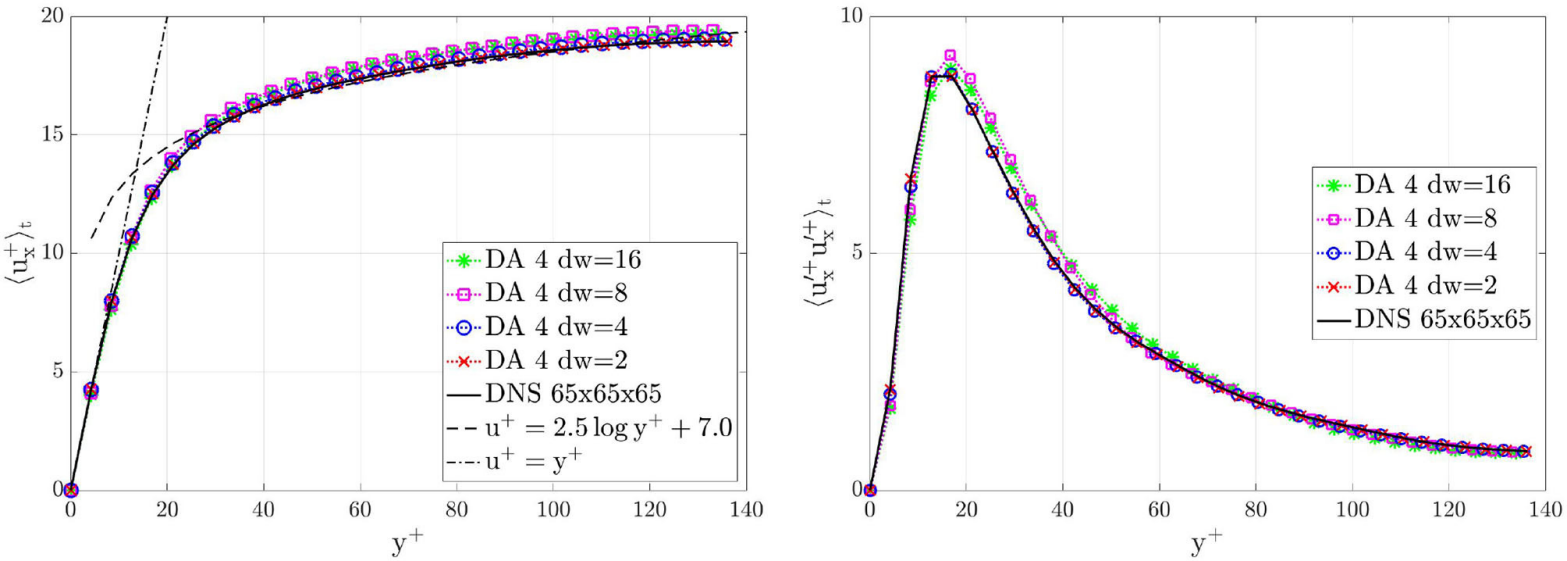
Turbulent channel flow: comparison between DA predictions obtained by using data acquired from window 4 with different downsamplings and ground truth on a line parallel to the *y*-axis and centered in the *z*-direction, i.e., *z* = π*h*, for *x* = 3π*h*. Left: stream-wise mean velocity profiles 〈*u*_*x*_(*x* = 3π*h, y, z* = π*h, t*)〉_*t*_. Right: RSS profiles ${\langle u_{x}^{\prime }\left(x=3\pi h,y,z=\pi h,t\right)u_{x}^{\prime }\left(x=3\pi h,y,z=\pi h,t\right)\rangle }_{t}$. Results are plotted in wall units and are shown for the bottom half part of the channel, i.e., *y* ≤ *h*.

Figure 11 shows the comparison between DA prediction and ground truth of the velocity profile ${\langle u_{x}^{+}\left(\vec{x},t\right)\rangle }_{t}$ profile and the RSS profile ${\langle {u\prime }_{x}^{+}\left(\vec{x},t\right){u\prime }_{x}^{+}\left(\vec{x},t\right)\rangle }_{t}$ on a line parallel to the *y*-axis and centered in the *z*-direction, computed after 4,000 time units. Downsampling the data increases the magnitude of the covariance **r**_*n*_ such that the filter will trust in them less than in the forecast solution given by the numerical solver. The recovery of the log law and the RSS peak predictions slightly deteriorate for higher downsamplings. Even though the unperturbed initial conditions of the flow would not have shown a transition to turbulence without the filter forcing, the boundary conditions are able to maintain the turbulence. After the numerical transient, the loose of accuracy due to the down-sampled data is compensated by the numerical solver. This is an important result because it means that the filter is able to give the correct importance to the two different sources of information.

### 3.3. Pulsatile Turbulent Flow Downstream a Bioprosthetic Transcatheter Aortic Valve

Here, the configuration setup for a pulsatile turbulent flow past a self-expandable Transcatheter Aortic Valve (TAV) with a heart-rate equal to 70 [beat/min] is investigated in the aortic region downstream the valve. The positive *y*-direction define the stream-wise direction, while the *x*- and *z*-direction are the span-wise ones. The Reynolds number is set to 4, 900, based on the nominal diameter *d* = 23 [mm] of the TAV, on the peak (systolic) velocity *U*_*y*_ = 1 [m/s] and on the kinematic viscosity ν of the fluid. In this case, no ground truth is available but only experimental data. Moreover, blood flow modeling uncertainty has to be taken into account.

#### 3.3.1. Data Acquisition

The generation of the data ${\vec{d}}_{v;\left(s\right),\phi }$ to be used in the DA predictions has been provided by an experimental setup. The TAV was implanted in a quasi-stiff aortic silicon phantom of the aortic root and integrated in a hydraulic pulse duplicator. Tomographic Particle Image Velocimetry (Tomo-PIV) (43, 44) was used to reconstruct the three-dimensional flow fields in the region of interest. The pulse period *T* = 60.0/70 [s] has been divided in $N_{\Phi }^{exp}=22$ intervals as described in the Table 1.

**Table 1:** Pulsatile turbulent flow downstream a bioprosthetic heart valve: division of the pulse period *T* in $N_{\Phi }^{exp}=22$ intervals and $N_{\Phi }^{num}=66$ intervals.

** $\phi_{i}^{exp}$ **	**1**	**2**	**3**	**4**	**5**	**6**	**7**	**8**	**9**	**10**	**11**	
$t_{\phi_{i}^{exp}}$[s]	0.05	0.06	0.07	0.08	0.09	0.10	0.11	0.12	0.13	0.14	0.15	
$\phi_{i}^{exp}$	**12**	**13**	**14**	**15**	**16**	**17**	**18**	**19**	**20**	**21**	**22**	
$t_{\phi_{i}^{exp}}$[s]	0.16	0.17	0.20	0.25	0.30	0.35	0.40	0.45	0.50	0.60	0.70	
$\phi_{i}^{exp}$	**1**			**2**	…	…	**21**			**22**		
$\phi_{i}^{num}$	**1**	**2**	**3**	**4**	…	…	**61**	**62**	**63**	**64**	**65**	**66**
$t_{\phi_{i}^{num}}$[s]	0.05	0.0533	0.0566	0.06	…	…	0.60	0.633	0.666	0.70	0.769	0.838

*Numerical time intervals have been obtained by refining by a factor of 3 the temporal resolution of each time interval of the experimental data*.

The experimental grid domain has a size of 35 [mm] × 50 [mm] × 35 [mm] and consists of 42 × 59 × 42 voxels. Each voxel-and-phase-averaged data has been computed by using 24 repetitions of the heart-pulse, and the covariance of the error of the PIV data results from a phase averaging of instantaneous voxel data fields taken at 24 pulses. Outside the region of interest, the data (velocity and corresponding uncertainty) have been set to zero in order to replicate the presence of the aortic walls. Of course there are additional errors due to the PIV methodology itself which are not considered in the present investigation.

#### 3.3.2. Comparison Between Experiments, Direct Numerical Simulation and Data Assimilation Prediction

Two simulations have been performed for a time equal to 120*T*: a DNS where the experimental data are used only to impose inflow and wall boundary conditions; and a DA prediction where the experimental data are used also to filter the forecast flow field by using the DA methodology. The pulse period has been divided in $N_{\Phi }^{num}=66$ intervals obtained by refining by a factor of 3 the temporal resolution of each time interval of the experimental data, as shown in the Table 1.

**Figure 12:**
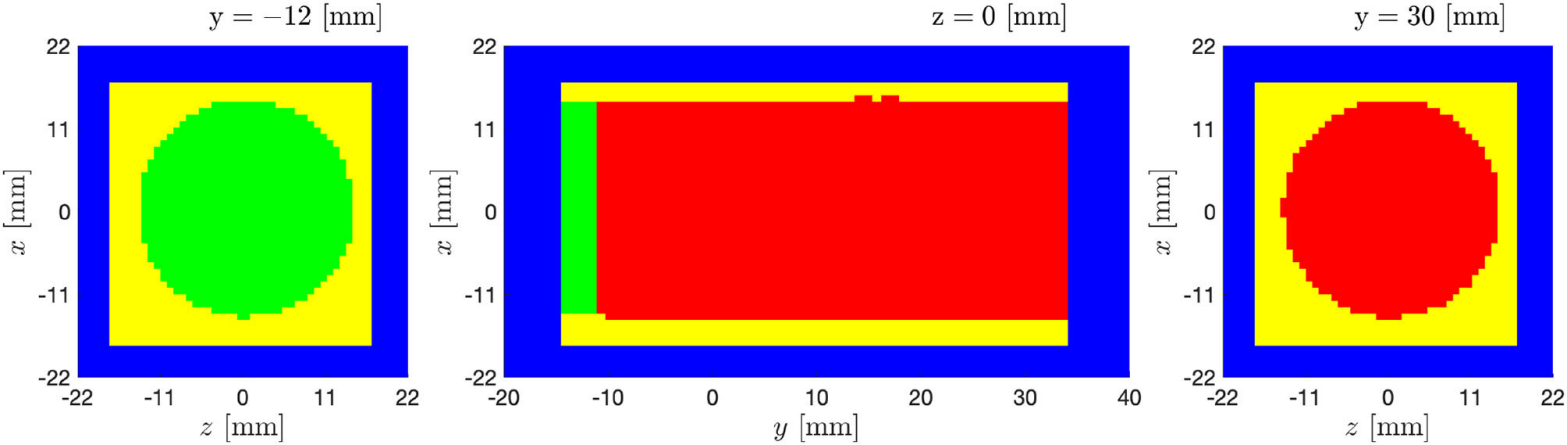
Pulsatile turbulent flow downstream of a transcatheter aortic valve: Numerical setup. The experimental grid is overlapped onto the computational domain (blue). The wall boundary conditions are imposed in the yellow region which includes data masked in the experiments; The inflow boundary conditions are imposed in the green region; DA is performed in the red region.

The computational grid used for both DNS and DA prediction has been obtained by refining the experimental grid by a factor of 2 in each direction and then adding an external padding equal to at least 10% to each side of the three-dimensional domain. The final size of the computational grid domain is 44 [mm] × 60 [mm] × 44 [mm] and consists of 105 × 145 × 105 grid nodes. Mapping the voxel-grid onto the computational grid leads to *g*_*v*_ = 3 × 3 × 3 grid-nodes lying inside the corresponding voxel. We use periodic boundary conditions for the full computational domain with padding. In both simulations, the presence of the aortic wall and the physical inflow conditions are enforced *via* the fringe forcing method (cf. Section 2.4). The experimental data forced to zero outside the region of interest and the lowest 5 *y*-plane slices data are used as desired values $\vec{U}$ of the fringe forcing applied in order to impose wall and inflow boundary conditions, respectively. Since the inflow is time dependent, it is not appropriate to use a constant value for λ in the Equation (41). Here, a time-dependent λ has been chosen such that it guarantees the stability condition of the Runge–Kutta time-advancement scheme:


(46) $$\begin{array}{l} \lambda \left(RK_{substep},\Delta t\right)=\frac{1.0}{\alpha \left(RK_{substep}\right)\Delta t}, \end{array}$$

where Δ*t* is the numerical time step and α is the first Runge–Kutta coefficient for each Runge–Kutta substep *RK*_*substep*_. The inflow boundary conditions for the intermediate numerical time-steps have been imposed by linear interpolation in time of the voxel data. The bulk flow voxel data are used only to perform DA prediction. The initial flow field is set to zero. A sketch of the configuration can be seen in Figure 12.

**Figure 13:**
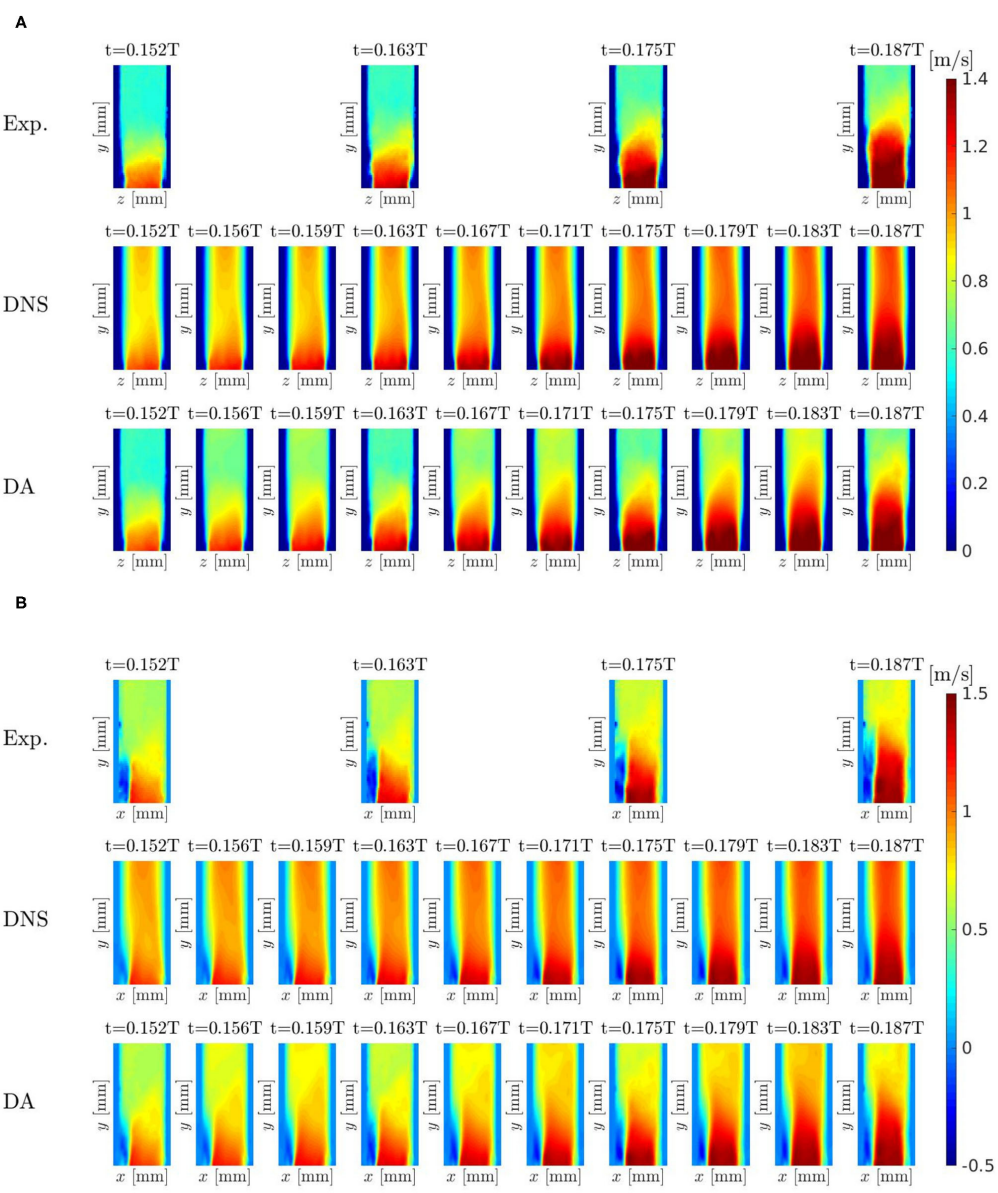
Pulsatile turbulent flow downstream of a transcatheter aortic valve: comparison between experiments, DNS, and DA prediction of velocity field ${\langle u_{y}\left(\vec{x},t_{\phi }\right)\rangle }_{\phi }$ for *x* = 0 **(A)** and for *z* = 0 **(B)**.

The results obtained by the experiments, DNS and DA prediction have been compared to evaluate the capability of the DA methodology to enhance the spatial and temporal resolution of the observations and to increase the reliability of the characteristic flow patterns captured from the numerical solver. Herein, the systolic phase-averaged velocity ${\langle u_{y}\left(\vec{x},t_{\phi }\right)\rangle }_{\phi }$ obtained by the Tomo-PIV experiments, DNS and the DA prediction are shown in Figures 13A,B for *x*-plane and *z*-plane, respectively.

The DNS and DA prediction present an enhancement in time resolution with respect to the experimental data. The boundary conditions extracted by the experimental data drive the flow in the intermediate iterations between two subsequent applications of the filter. This is an important result because it has been possible to simulate the cardiovascular turbulent flow downstream the heart valve numerically without the need for Fluid-Structure-Interaction methods.

In the DNS, the results show a global overestimation of the velocity field. This is due to boundary condition uncertainty and blood modeling uncertainty. The DA prediction is affected by the same boundary condition and blood modeling uncertainties but the DA prediction strongly reduces the overestimation of the DNS velocity field. It means that the use of the DA methodology has improved the reliability of the velocity field results. The three leaflet jet coming from the bioprosthetic valve seems to be better defined in DA prediction (see, e.g., *t* = 0.175*T* slice, in Figure 13A). In the lower part the back flow near the left wall, the DA prediction present a smoother flow field (see Figure 13B). This is due to the higher capability of the numerical solver to investigate that region which is, on the contrary, a more difficult challenge for the experimental tool. It is noteworthy that the DA prediction presents a smaller reverse flow region: the filter trust more in the numerical solver and consider the bigger reverse flow region present in the experiments too uncertain with respect to the forecast solution. Moreover, the DA methodology filters the information coming from those voxel data near the wall that are evidently affected by a tool acquisition error. This error is not visible anymore in DA prediction.

**Figure 14:**
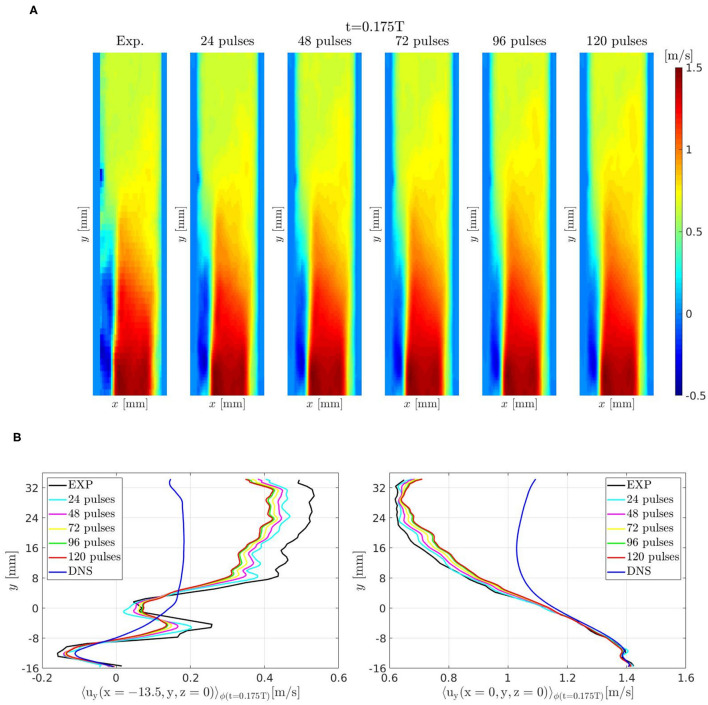
Pulsatile turbulent flow downstream of a transcatheter aortic valve: **(A)** velocity field 〈*u*_*y*_(*x, y, z* = 0, *t*_ϕ_ = 0.175*T*)〉_ϕ_ obtained by the DA algorithm for increasingly larger number of pulses. **(B)** Velocity profiles along a line parallel to *y*-axis and crossing the reverse flow region, *x* = −13.5 mm and *z* = 0, (left) and along the centerline, *x* = *z* = 0, (right).

Figure 14A shows the evolution of the flow field for increasingly larger number of pulses performed by the DA algorithm. Velocity profiles from DA prediction, DNS, and experiments are compared in Figure 14B along two lines parallel to *y*-axis. The results show that the flow field prediction has reached a good convergence after 120 pulses.

The finer spatial and time resolution obtained in the DA prediction enhanced the flow field of this specific configuration. This is an important and promising result for future applications of the proposed DA methodology on *in vivo* data from Flow-MRI of patient-specific configurations.

## 4. Discussion

In the present manuscript, a new DA methodology has been presented. The robust theoretical background of the (S)EnKF approaches has been applied to pulsatile and turbulent flow configurations. The different ensemble-averaging approaches, that were presented here, show how the study of turbulent flows can be reduced to the definition and the subsequent investigation of the most important statistical properties of the flow, such as mean velocity and the associated fluctuations covariance.

In the context of turbulent flows, the proposed SEnKF-based methodology states as hypothesis that the “true” state-of-system is the mean velocity field. Turbulent fluctuations are deviations from the ensemble-averaged quantities. This leads to the formal equality between turbulent fluctuations and the observation error ${\vec{r}}_{n}$ required in the derivation of a SEnKF.

In the derivation of our DA methodology, the definition of the states of the ensemble allows us to treat the forecast error ${\vec{q}}_{n}$ in the proper way: this error has zero mean and the contribution of the covariance of the numerical error ${\vec{q}}_{n_{NUM}}$ will be negligible compared with the one of the turbulent error ${\vec{q}}_{n_{TUR}}$ and modeling error ${\vec{q}}_{n_{MOD}}$. In the first two test cases (sections 3.1, 3.2), the model is well defined and therefore ${\vec{q}}_{n_{MOD}}$ is zero. In the last test case (section 3.3), DNS and DA predictions are affected by the same ${\vec{q}}_{n_{MOD}}$. Therefore, only the turbulent error is relevant in the present study.

The choice of the ensemble of states-of-system in the case of pulsatile and turbulent flows allows one to filter the forecast solution at each time *t*_*n*_ with the corresponding data flow field; this yields to an additional but constant computational cost for each pulse interval.

From a numerical point of view, filtering the forecast solution with a forcing term only in nonconsecutive time-step iterations could be seen as forcing the flow field with a temporal Dirac δ function. This would lead to numerical oscillations in the intermediate iterations between two filtering iterations and to a local non-smooth advancement in time of the numerical solution.

First, the DA methodology has been validated vs. an unsteady flow past a cylinder confined in a channel using data acquired from previous numerical simulations. In the DA predictions, the presence of the cylinder was not taken into account by using any boundary conditions but nevertheless the reconstruction of the wake downstream the cylinder has been successfully reconstructed by filtering the forecast solution with the data. The test shows that the DA method, and in particular the proposed choice of the ensemble of states-of-system, copes with unsteady flow field presenting a periodicity in time.

Second, the methodology was extended to turbulent flow applications. The statistically-steady configuration of the wall-bounded turbulent flow shows that the turbulent features can be reconstructed by using the data even though the numerical simulation is initialized with a laminar flow which would not exhibit a transition to turbulence by itself. The effect of the different data configurations on the bulk flow and on the walls has been investigated. Downsampling the resolution of data acquisition leads to a loss of accuracy in the DA prediction that is limited to 5%.

Finally, the methodology has been applied to pulsatile and turbulent configuration of a flow past a bioprosthetic heart valve. The data of a pulsatile turbulent flow past a TAV, acquired by a Tomo-PIV technique have been used to reconstruct the wake in the aorta in a computational simulation with higher spatial and temporal resolution. The test-case shows how the DA methodology deals with FSI applications even though no structural modeling is required. The results show the capability, the robustness and the accuracy of the proposed methodology to cope with realistic configurations of biomedical applications.

The presented results show that the filter is capable of interpreting the accuracy and reliability of the data with respect to the numerical solution giving locally a different weight to the two different sources of information. A bigger amount of data, although with greater uncertainty, allows to lead the solution of the DA method closer to the true-state of the system. This is useful in heart valve applications where the data acquired by experimental investigations or through 4D Flow MRI techniques suffer from lower accuracy near the walls; these noisy data are filtered and the higher accuracy of the numerical solver will return a better description of the flow field in such regions. On the other hand the available data of the specific-patient configuration will increase the accuracy of the numerical solution in the bulk flow if compared with the solution obtained by using a FSI solver employing a simplified morphology of the aortic valve and aortic root.

A first limitation of the methodology is that we consider the covariance across different voxels to be zero. This is equivalent of assuming that the autocorrelation length is less than the voxel size *h*. This limitation might be overcome solving with higher computational cost the analysis step by using the global matrices **H**, **P**^*f*^ and **R** instead of **h**, **p**^*f*^ and **r**. A second limitation of the methodology is represented by the definition of the matrix **D** which is required to ensure positiveness of covariance matrix **p**^*f*^. From a theoretical point of view **D** influence disappears for increasingly large ensembles, but the choice of its value strongly affects the convergence history of the DA prediction. For **D** too high, the DA prediction will require a larger number of cycles in order to reach a converged solution for both mean velocity and covariance. Moreover, a large value of **D** reduces the physical meaning of the matrix **p**^*f*^ and this will influence the sensitivity of the filter. On the other hand, for **D** too low the forecast solution would be considered closer to the “true” state-of-system by the filter even though it cannot be stated a priori. In conclusion, the value of **D** has to be chosen high and run the DA prediction for the required number of cylces until convergence is reached. For this reason, in the DA prediction of the pulsatile turbulent flow downstream a bioprosthetic heart valve, 120 pulses (5 times the number of repetitions available by the experiments) have been enough to enhance the description of the mean flow fields with respect to the observations, but have not produced a significant improvement of the *RSS* fields. The investigation of the *RSS* fields requires to collect a larger ensemble for both matching the classical requirements on the ensemble size of turbulent statistics and fully overcoming the influence of **D**. The sensitivity of the proposed methodology to the matrix **D** and the size of ensemble needed for convergence in these cardiovascular applications in the ascending aorta will be further investigated in order to estimate the computational cost required to enhance *RSS* fields. A further limitation is that synthetic experimental data have been created without adding any Gaussian error after spatially averaging inside the voxel, even though this has been previously done in literature. This choice has been made here, because we want to focus our investigations on the effect of turbulent fluctuations (considered as measurement noise). Additional noise may pose additional problems to the described method, in particular, because the real noise in MRI data has a non-Gaussian character. In practice, 4D flow MRI quantification would require more complex methods in order to quantify the effects of these hardware source of errors. Specific methods have been developed for creating synthetic 4D flow MRI data from raw Phase Contrast MRI data to better assess turbulent features, e.g., turbulence intensity and TKE (45–50), and for completely excluding the effects of the hardware source errors by generating synthetic MRI data fields (51).

In conclusion, the results show that the method is promising for future use with *in* *vivo* data from 4D Flow MRI.

## Data Availability Statement

The raw data supporting the conclusions of this article will be made available by the authors, without undue reservation.

## Author Contributions

DD and DO wrote the manuscript and reviewed the manuscript and designed the data assimilation method. DD implemented the data assimilation method in the numerical solver and performed the simulations. DO supervised the scientific work. Both authors contributed to the article and approved the submitted version.

## Funding

This research was supported by the Platform for Advanced Scientific Computing (PASC, http://www.pasc-ch.org) under the HPC-PREDICT project (https://www.pasc-ch.org/projects/2017-2020/hpc-predict).

## Conflict of Interest

The authors declare that the research was conducted in the absence of any commercial or financial relationships that could be construed as a potential conflict of interest.

## Publisher's Note

All claims expressed in this article are solely those of the authors and do not necessarily represent those of their affiliated organizations, or those of the publisher, the editors and the reviewers. Any product that may be evaluated in this article, or claim that may be made by its manufacturer, is not guaranteed or endorsed by the publisher.
